# Hybrid methods in flood inundation modeling: a systematic review

**DOI:** 10.1007/s11069-026-08078-w

**Published:** 2026-04-02

**Authors:** Manimeldura Devki Devanga Perera, Athanasios Angeloudis, Adil Siripatana, Lindsay Beevers

**Affiliations:** Institute for Infrastructure and Environment (IIE), School of Engineering, University of Edinburgh, Edinburgh, EH8 9YL UK

**Keywords:** Data-driven, Hybrid models, Flood inundation, Evaluation metrics, Benchmarking

## Abstract

Flooding is a destructive natural hazard that is exacerbating due to climate change, causing significant socio-economic losses. Flood inundation models are critical tools in the mitigation and management of such hazards. Conventional standalone process-based models can be limited in real-time applications due to their computational intensity and the trade-off between accuracy, resolution and speed. Data-driven (machine learning) models offer an alternative route as surrogate models, promising computational efficiency, however, are noted to have limitations of high data-dependency and a black-box nature. Hybridization, implemented in machine learning models to enhance strengths and limit tradeoffs has been shown to increase physics awareness, real-time applicability, and adaptability. This paper conducts a systematic review of state-of-the-art research on hybrid flood models, and presents a definition of hybridization. A classification of hybridization techniques into enhancing inputs, structure and processing is proposed. Sequentially, common metrics used to evaluate flood models are classified into accuracy and speed-based metrics, and the usefulness of hybridization is discussed. An extensive benchmarking framework is proposed for the comparison of models before application, based on quantitative classification of the model and standardized datasets and tests. Finally, a way forward is proposed in the context of physics informed machine learning for flood inundation modeling.

**Supplementary Information:**

The online version contains supplementary material available at 10.1007/s11069-026-08078-w.

## Introduction

Flooding is one of the most common natural hazards (Schmid and Leandro 2023), which are more widespread and frequent and with the increase in rainfall intensity due to climate variability (Adriano et al. 2023). Floods can adversely impact livelihoods, infrastructure, and socioeconomic systems (Beevers et al. 2020; Panahi et al. 2022), and can induce significant changes in floodplain hydro-morphodynamics which respond rapidly to extreme hydrological disturbances. These trigger alterations in river morphology, sediment transport, mineral leaching, and have impacts on flora and fauna (Panahi et al. 2022). Early warning systems are a cost-effective solution for mitigating flood losses (Yang et al. 2016), and effective real-time flood prediction models are crucial in the development of accurate flood maps for sustainable flood management and spatial planning (Adriano et al. 2023; Schmid and Leandro 2023). Flood inundation maps determine the extent of a flood during or after occurrence and represent flooded and non-flooded areas. In turn, flood susceptibility maps determine the tendency of flooding in an area based on its physical characteristics. Similarly, flood hazard maps measure the depth and extent of the water in an area and consider different return periods of the flood and the probability of flooding (Bentivoglio et al. 2022). Forecasting systems are expected to be accurate, reliable and timely to produce inundation maps that represent the area of flooding and complement river discharge hydrographs well (Zanchetta and Coulibaly 2022).

However, the dynamic, stochastic and variable nature of climate change, hydrological processes, and river systems make deterministic modeling that relies on single sets of boundary data inadequate for capturing the complexities of flood depths, velocities, and inundation extents (Mosavi et al. 2018; Beevers et al. 2020). For decades, computational models have been used in flood simulation (Marshall et al. 2017) where high performance models are required for rapid and accurate prediction to mitigate increasingly severe flood hazards (Wang et al. 2024). Physics based and distributed numerical models that are widely used for flood modeling and are based on are rooted in complex mathematical equations, requiring substantial hydro-geomorphological datasets and entailing high computational costs. This makes their real-time applications difficult and limits their use in early warning systems (Chang et al. 2014; Yang et al. 2016; Wang et al. 2024). Similarly, statistical models that assimilate measured climate indices and hydro-meteorological conditions (e.g., autoregressive moving average, multiple linear regression, etc.) face limitations such as inadequate robustness, lower accuracy, complexity, and high computational cost (Mosavi et al. 2018). To overcome these challenges, more coupled modeling techniques capable of adaptively extracting complex input–output relationships of a domain must be implemented (Chang et al. 2014). The drawbacks of standalone conventional models have encouraged the research on advanced data-driven surrogate models implementing artificial intelligence (AI) and machine learning (ML) that can balance accuracy, speed and data requirements, to analyze hydrometric data for patterns (Wang et al. 2024) in flood inundation modeling. Depending on the ML techniques used, they allow the formulation of nonlinearity, are easier to implement, have lower computational costs, and reduced complexity (Mosavi et al. 2018; Kabir et al. 2020). Mosavi et al. (2018) noted that ML models used in flood prediction can be separated into short-term predictions (hourly, daily and weekly), and long-term predictions (longer than a week). These methods can be separated into single and hybrid architectures. Flood models can be evaluated based on the accuracy, efficiency, reliability, robustness, consistency, generalization and timeliness.

Despite their advantages, ML methods also have considerations that must be taken into account, such as their training requirements, data-dependent output, their black-box nature (internal processes lack basis in physics), and their ‘generalization problem’ (limited performance in unseen data). Studies report improvements in ML methods by novel hybridization techniques that can result in higher prediction accuracy, lower uncertainty, better robustness, and more realistic flood models compared to standalone models (Mosavi et al. 2018). Hybridization aims to combine the strengths of multiple techniques with ML architectures for improved performance. Although hybrid modeling has gained popularity in flood simulation, with numerous studies affirming their functionality and merit, the term ‘hybrid flood models’ remains ambiguous and loosely defined. Kurz et al. (2022) defines ‘hybrid models’ as combinations of first-principles-based models with data-driven model architecture to improve model qualities and explainability. The definitions and applications of hybrid models in flood simulation vary across research, encompassing hybrid data inputs, hybrid model structures, and hybrid processing methods. Various novel flood inundation models have been developed through hybridization, typically for better performance and faster implementation. However, limitations exist in these model structures such as the need for retraining, unsatisfactory generalizability, and semi-blackbox natures. Due to the widely varying nature of the structure and implementation of these models, benchmarking becomes a very important phase of building and in selecting a model for a targeted application. The lack of benchmarking leads to ambiguous evaluation metrics that are inadequate and too vague to be used in comparison across models. Although research on hybrid flood modeling is emerging rapidly, the structure and performance outcomes of these models vary considerably, highlighting the need for further standardization and evaluation.

The aim of this paper is to systematically review the research on hybrid flood inundation models and present a way forward in benchmarking. The main objectives were structured as follows;

1. Explore the limitations of standalone hydrodynamic models and common ML architectures used for flood inundation modeling that consequently drive the research on hybrid models.
2. Define ’hybridization’ and analyze the techniques applied in creating a hybrid flood model.
3. Examine the common performance metrics used in evaluating hybrid flood models specifically used in flood inundation modeling and present a benchmarking framework for model comparison.
4. Critically analyze the success of current ‘hybrid flood models’ and suggest a way forward.

## Methods

**Fig. 1:**
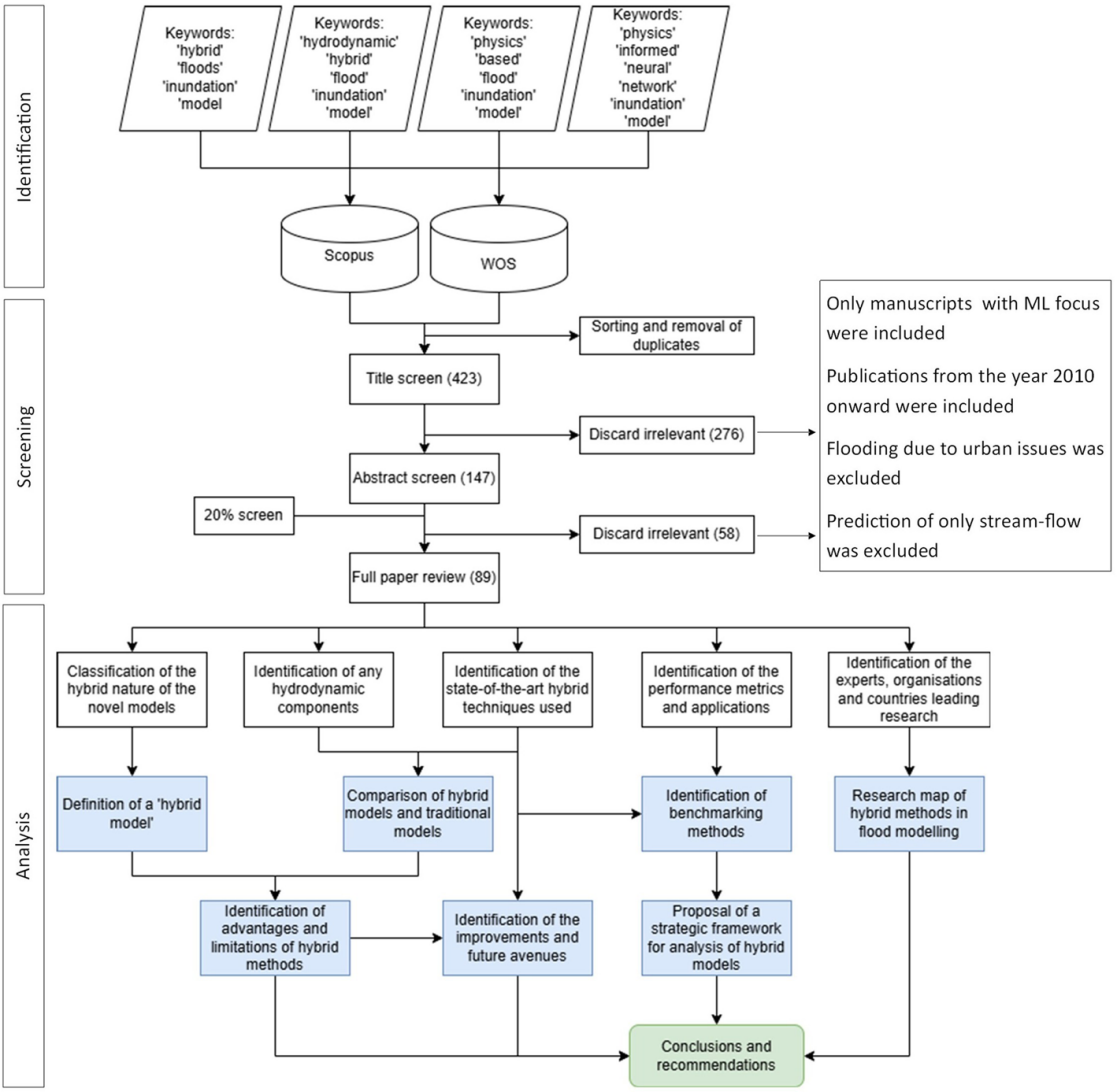
Methodology of the selection and systematic review of literature

We started with a keyword search (Mosavi et al. 2018; Bentivoglio et al. 2022) to identify relevant literature in the Scopus and Web of Science (WOS) databases, using search terms that best represent the latest research on hybrid models in flood inundation modeling (Fig. 1). The keywords and combinations used for the search (last search in May 2025) were ’hybrids + floods + inundation + model’, ’hydrodynamic + hybrid + flood + inundation + model’, ’physics + based + flood + inundation + model’, ’physics + informed + neural + network + inundation + model’. The presence of the keywords were searched in the title and the abstract of the papers. Manuscripts available and published online, in the english language were considered. The search terms ( Fig. 1) resulted in 97, 28, 22, 70, 6 documents from the Scopus database and 104, 27, 45, 180, and 6 documents from the WOS database, respectively. Duplicate results from the database were filtered out and the literature was reduced to 423 entries, comprising 360 journal papers, 52 conference papers, 6 review papers, and 2 book chapters. The documents were screened by title and then abstract, with the inclusion and exclusion criteria that only manuscripts focusing on ML were considered, flooding due to urban issues such as drainage failure were not considered, prediction of only stream-flow was excluded, and only publications from the year 2010 onward were considered to keep the research current. Finally, 79 journal articles, 7 conference articles, 3 review articles, and 1 book chapter were selected for the full-text review. All authors performed a 20% screen for quality assurance, with 95% agreement; the differing articles were discussed and the screening process was rechecked. The classification of the documents based on year and type, before and after screening was used to identify patterns in research trends geared towards hybrid flood models.

This paper presents a detailed review focused on the research objectives outlined in Sect. 1. Section 3.1 plots and discusses the number of publications on hybrid flood models per year and the global distribution of the author institutions conducting this research. Section 3.2 explores the up-and-coming ML methods used in flood inundation modeling, their advantages and their limitations. The techniques used in building a hybrid model are classified in Sect. 3.3 and a standard definition of hybridization is suggested. Section 3.4 presents the diversity of metrics used in assessing the models of the literature reviewed, and highlights the need for benchmarking. Section 4.1 revists the limitations of standalone models and examines if hybrid models are advantageous and notes the limitations. Section 4.2 presents a benchmarking framework that can be used to evaluate and compare hybrid models with each other, and standalone hydrodynamic and ML models. Section 4.3 suggests a way forward by incorporating physics into ML models.

## Results

### Bibliography analysis

**Fig. 2:**
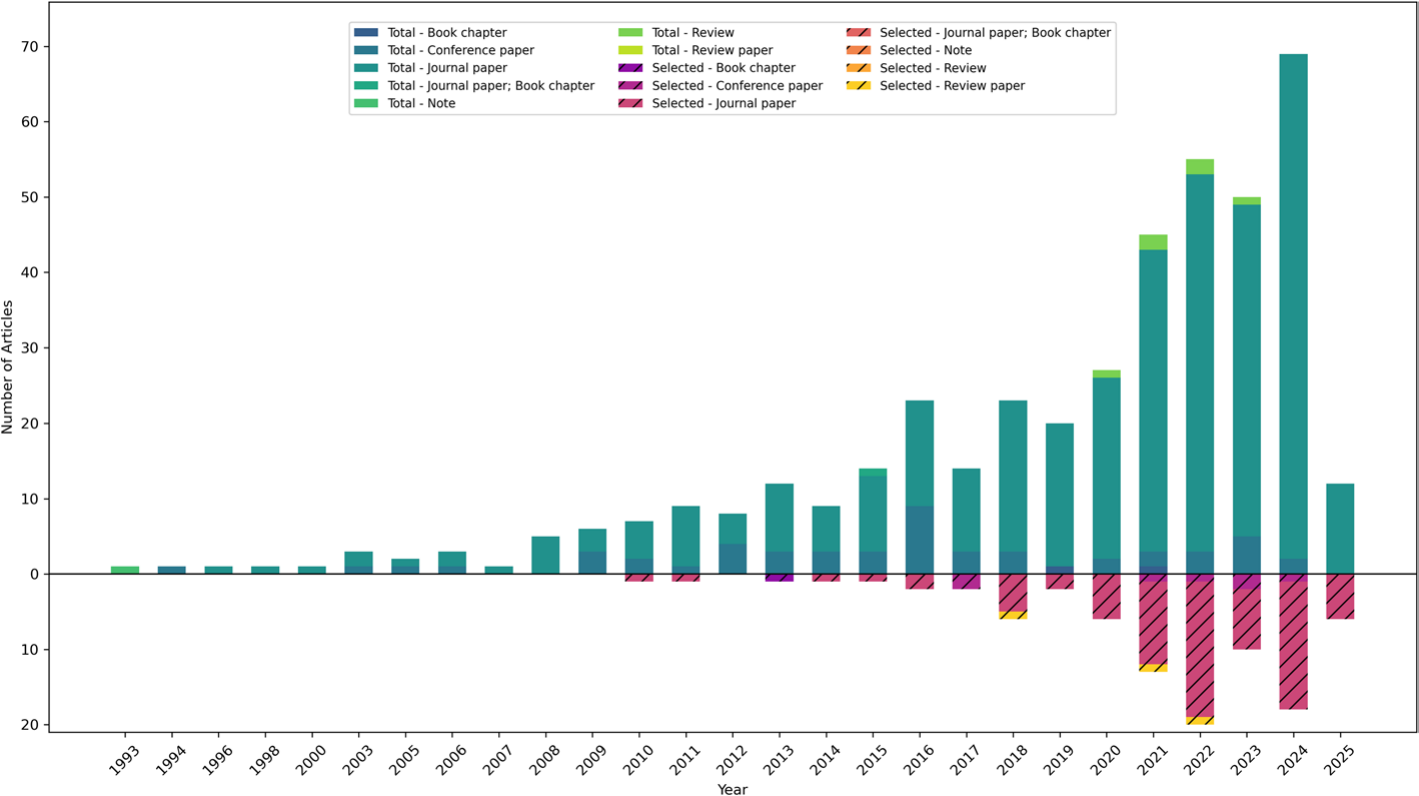
Number of publications on hybrid flood inundation models per year from the keyword search on WOS and Scopus (Top chart shows the total number of articles from the search, bottom chart shows the selected articles for full text review after screening)

Figure 2 plots the total number of articles from the SCOPUS and WOS search, and the articles selected for full-text review sorted by the year of publication. An increasing trend of the research published over the years was observed for both the total and selected articles, indicating an increasing interest in hybrid flood inundation models. The year 2025 is negated in this discussing this trend, since articles published in only part of the year were considered.

**Fig. 3:**
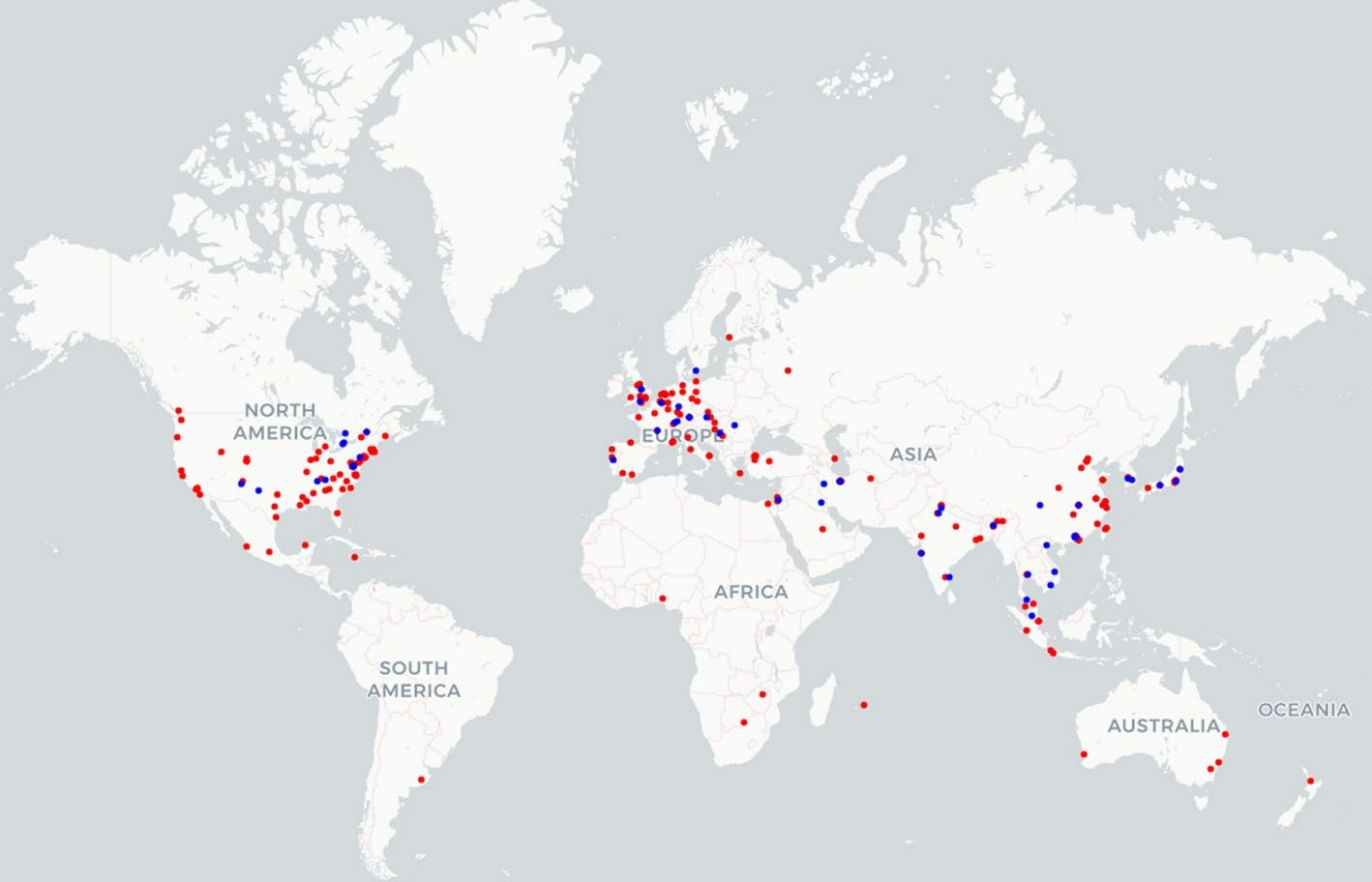
Map of the institutions of authors conducting research on hybrid flood inundation models (red - total number of articles from searches; blue - articles selected for the full text review)

Figure 3 shows the research map of the institution geography (derived by author affiliation) that determines the distribution of the research on hybrid models carried out globally. It was observed that the majority of institutions were located across Europe, North America, China and India.

### Common data driven (ML) methods used in flood inundation modeling

Standalone ML models are an emerging avenue in flood modeling and can assimilate climate indices, hydro-meteorological parameters, and identify input–output patterns for rapid prediction (Xu et al. 2023; Xie et al. 2021; Feng et al. 2023). Mosavi et al. (2018) notes that several studies show their success in flood prediction, and that in certain cases they outperform conventional approaches. Data-driven surrogate ML models can aid flood forecasting by circumventing intensive computation and returning predictions within a shorter time; these can be classified under lower-fidelity models that focus on dominant physical processes. Surface response models that are more comprehensive can improve precision for rapid flood modeling. Deep Learning (DL) models aim further to automatically discover representations needed for detection and classification of raw data and learning hidden patterns for improved performance. DL models can have increased accuracy and flexibility, less bias, and can be used for real-time applications due to their rapid simulation capacity (Bentivoglio et al. 2022). In this paper, we generalize both ML and DL methods and refer to both as ML methods for simplification. The general process of building a data-driven model includes the collection of input data, pre-processing, building the architecture, training and optimization, validation, re-training and testing before application.

Despite the many advantages of ML and DL models, common challenges are highlighted with regards to data dependency, black-box nature, lack of physics interpretation, and the limited generalizability. The term blackbox is used as the the mapped relationships lack basis in physical laws and the inner working is not transparent to the user. Although faster, this is a prominent disadvantage compared to numerical models based on equations. The generalization capabilities of standalone ML models are limited, as they require retraining for different cases. Simple ML models have less prediction ability in spatially distributed cases, and advanced modeling strategies are needed to model complex floods (Dang et al. 2024). This implies the need for improving ML models to enhance strengths and reduce limitations and tradeoffs between accuracy and speed; this is the aim of hybridization.

Table 1 summarizes the different ML architectures noted in the reviewed literature. Short descriptions are given for each type of architecture. The selection of the ML method for building a model depends on the input data available and the target outputs. The level of accuracy and prediction speed required are also factors to be considered.

**Table 1:** Common data driven (ML) methods used in hybrid flood inundation modeling

Architecture	Description
Support Vector Machine (eg. Panahi et al. (2022))	Supervised learning machines based on statistical learning theory, and are robust, efficient architectures with good generalization and performance, suitable for linear and nonlinear tasks. Can require optimization and can be time consuming, have high computational cost, unrealistic outputs, and semi black-box nature
Decision Tree (DT) (eg. Ngo et al. (2021))	Effective supervised algorithms that split the dataset into branches based on decision rules learned from input features. Simple and interpretable, are suitable for nonlinear modeling, and have fast inference. However, can be prone to overfitting
Adaptive Neuro Fuzzy Inference System (ANFIS) (Ngo et al. 2018)	Based on the Tagasaki-Sugeno fuzzy modeling technique with high learning capability and are suitable for nonlinear simulations. Has easy implementation and good generalization, however depends on input quality and may require extensive parameter optimization
Extreme Machine Learning (ELM) (Ebtehaj and Bonakdari 2022)	Single-layer feedforward neural networks with fast learning speed, high accuracy, and good robustness. The random determination can affect generalizability, forecasting accuracy, and the existence of outliers
Neural Networks (NN) (Jhong et al. 2018)	Versatile and efficient architectures inspired by the human brain and designed to recognize patterns, learn relationships, and make predictions from data. Have high fault tolerance, can make accurate approximations and have acceptable generalization and speed. Is black-box in nature, can be prone to overfitting and may require large datasets
Multi Layer Perceptron (MLP) (Kabir et al. 2021)	Utilizes supervised learning, is simple, can map complex non-linear relationships, and is versatile. Can be black-box in nature and can be prone to overfitting
Wavelet Transform (WT) (Rahmati et al. 2020)	A signal processing technique used to analyze data at multiple resolutions or scales, and extracts information by analyzing variations in time-series. Can handle non-stationary and non-linear data and reveal hidden patterns. Can have increased computational cost and complex interpretation
Nonlinear Autoregressive with Exogenous Inputs (NARX) (Zanchetta and Coulibaly 2022)	Is a simple recurrent model structured as a feed forward neural network and can capture temporal dependencies, is suitable for non-linear dynamic systems and has good sequential prediction. But training may be computationally intensive, is sensitive to input lag selection and requires a well structured dataset
Gaussian Process (Fraehr et al. 2022)	Can predict complex non-linear relationships by assuming that the relationship between input and output follows a Gaussian distribution of functions. However, can be expensive for large datasets

### Definition and classification of hybridization

ML models can be combined with a variety of other techniques and resources such as Satellite aperture radar (SAR) data (Liu et al. 2019), numerical models (Li and Willems 2020), and even social media or crowd sourced data platforms (Sirsant et al. 2024) for better functionality and performance. A highlighted point is that is the integration of physical constraints that can increase accuracy and interpretation (Sun et al. 2023) of ML models by enabling the mapping of non-linear relationships between input/output variables without violating laws of physics. Such improvements can enhance ML models and compensate for limitations where the end goal is to create models with reduced tradeoffs, consequently leading to increased accuracy without needing high resolution data, good generalization, lead time and speed. Hybridization of numerical and ML models can have the desired effect, combining the strengths of both types, eventually leading towards accurate models with short-term applications. An ideal model would require minimal inputs, and have fast development, training and prediction speeds, easier implementation, lower computational cost and complexity, higher performance and accuracy, and could be used for both long, and short-term applications.

Although the reviewed literature explicitly states that each novel model presented is of a ‘hybrid’ nature, few define the term directly. Some studies have referred to the incorporation of physics into ML models, such as where Gao et al. (2024) recognizes hybrid models that combine physics-based models and data-driven techniques. Li and Willems (2020) highlights that hybrid models that integrate physical process knowledge in data-driven models are gaining attention for their potential to achieve a more robust performance beyond the training data set. Kurz et al. (2022) explicitly defines hybrid models as those that combine first principle-based models with data-based models into a joint architecture, supporting enhanced model qualities, such as robustness and explainability. Other definitions refer to the combination of two or more ML techniques. For example, Munawar et al. (2021) notes that hybridization is based on combining two or more ML techniques for predicting floods by creating systems that use the best features of different methods. Rahmati et al. (2020) defines hybridization as the integration of two or more ML methods, or of ML and more conventional methods, and/or soft computing ­techniques. The review paper by Mosavi et al. (2018) classifies hybrid models as those that combine more than one ML method. Others mention the amalgamation of different techniques or models in a broader sense. Xie et al. (2021) notes that hybrid models integrate the advantages of different individual models and are regarded as useful tools to capture the complex nature of a real-world system. while Habibi et al. (2023) adds that hybrid models improve predictive models with high dimensions more accurately. Rathnasiri et al. (2023) notes that hybrid approaches combine various data sources, such as sensor data, satellite imagery, and weather forecasts, with advanced analytical techniques, including ML and AI, to enhance flood risk assessment, early warning systems, and mitigation measures. Kurian et al. (2020) states that hybrid models combine the strengths of two or more modeling approaches. Hinge et al. (2024) notes that researchers have proposed the formation of hybridized models by optimizing the parameters of various ML models. Jeba and Chitra (2024) and Fares et al. (2023) note that hybridization of models by combining two or more approaches simultaneously or sequentially could take advantage of each method’s predictive ability and harness the strengths of each to overcome the limitations.

The literature reviewed present models that fall into one or more of these definitions, and therefore, the term ’hybridization’ needs to be broad enough to capture all these types of models. Following these ideas, we define a hybridization (in the context of ML used in flood inundation modeling) as a method of combining the strengths of standalone process-based (hydrodynamic) and ML models, to enhance the input, structure, or processing of the resulting hybrid model. It is agreed that hybridization aims to improve model performance in terms of physics incorporation, representability, accuracy, speed and generalizability, while decreasing limitations. It was noted that hybridization was centered around the modification of different points of a ML model structure. Hybridization can be done using various additions at the model input/training, architecture (structure), or the processing. Strategically combining the different methods of hybridization can result in more advanced models with improved qualities and applications, where the aim is building a hybrid model with reduced tradeoffs between accuracy and resolution.

Enhancing the input of ML models can be done by incorporating hydrodynamic models and satellite aperture radar (SAR) data to generate datasets for training and testing. These methods are implemented as an improvement to using only observed gauge data that may be spatially and temporally sparse. SAR data is usually available in most conditions and are continuous and abundant temporally and spatially in high resolutions (Liu et al. 2019; Panahi et al. 2022; Ngo et al. 2021). The significant advantage of SAR data lies in the rapid data collection from which ML models can learn feature patterns for real-time applications. However, data extraction can be time-consuming, and the large datasets need pre-processing. Self Organizing Maps (SOMs) are a common method in clustering data to provide visual insights (Chang et al. 2018), however, limitations still exist in the form of data dependability and the semi-blackbox nature as hydrodynamic equations are not incorporated. Using hydrodynamic models to generate data increases the ability of the model to map non-linear and complex relationships between input and output variables (Dang et al. 2024). Input data into the hydrodynamic model itself usually involves topographical data, meteorological data, geological data, geographical data, and anthropogenic data, and the output can be mapped in the forms of water depth, inundation extent and probability of occurrence, etc. to act as target references for data-driven models (Bentivoglio et al. 2022). Hydrodynamic models can generate clean datasets and capture extreme events. Although this method marginally accounts for the incorporation of physics (such as hydrodynamic equations) into models during training, retraining is required, data generation is slow, and models need calibration, often limiting real-time applications (Zanchetta and Coulibaly 2022; Schmid and Leandro 2023). The resultant hybrid model can only build input output relationships and inherits errors in datasets.

Changes to the structure of ML models can be advantageous in improving performance, accuracy and robustness of models. Optimization is applied for tuning hyperparameters such as learning rate, number of layers, batch size, dropout rate and kernel size (Xu et al. 2023). Optimization ensures faster and more stable training, and reduces computational waste and data requirements. However, there can be model overfitting and increase in black-box nature and complexity. Feature-informed models are integrated with important physical and topographical details (Schmid and Leandro 2023; Situ et al. 2025), and consequently, the computational waste can be reduced with the model focused on critical features. The realism, flexibility, and computational efficiency of the model can increase, however, the model data dependency and complexity can also increase with increasing resolution. Physics informed neural networks (PINNS) incorporate physical laws into ML models based on different architectures, by embedding physics (such as of the shallow water equations) in the form of partial differential equations, where the data loss is supervised using available observations, and the physics loss function enforces the governing partial differential equations using automatic differentiation (Donnelly et al. 2023). As a result, the black-box nature of the models are reduced as PINNs are built on equations ensuring that real-world hydrodynamic laws are applied, and this in turn increases the data efficiency and generalizability (Jhong et al. 2018). However, training PINNs can be computationally expensive since they are more complex than simple data-driven models.

Hybridization can be done by combining different ML structures to run separate processing functions, for example Xu and Gao (2024) used LSTM for predicting drainage outflows and CNN for predicting water depths. This method can compensate for the limitations of standalone ML models by selecting the best architecture to perform a specific task and combining strengths of the individual architectures used. Ensemble models can have increased accuracy, physics awareness, adaptability, robustness, and generalizability. However, these models tend to be data intensive, have increased black-box nature, and require careful calibration. Parallel processing can increase computational efficiency by dividing computational tasks into multiple processors or cores, leading to faster model training, evaluation and data handling. Parallelization can increase the scalability and real-time applicability and is advantageous in running complex models with detailed physics. However, it can be challenging to design efficient algorithms for parallelization to work well, and hardware knowledge is required. The data exchange can also cause lags and increase hardware dependency.

Ultimately, the goal of hybridization is to augment standalone models, and the optimal way of building a hybrid model would depend on the characteristics that need to be prioritized and the intended nature of application. For example, if the model needs to be used in a fixed domain, with no real-time applications, using data simulated by a hydrodynamic model to train a simple black-box data-driven model for low accuracy predictions may be more computationally efficient (Kabir et al. 2020). Each method and combination can have different benefits and shortcomings, and ultimately it is up to the user to decide which combination of methods works best for the purpose. The schematic of the hybrid model would also vary depending on the methods used, however, since these models are mainly centered around ML architectures, they will still retain a similar base structure with additional features. The effort required in different stages of model structures vary, in hydrodynamic models, high focus is given to the model setup and calibration, and to the model architecture and training in ML models. Hybridization aims to reduce the labor at different phases of building the models for more efficient implementation. Supplementary data gives an insight into the new hybrid models presented in literature categorized according to the hybridization techniques used (Table 6).

**Fig. 4:**
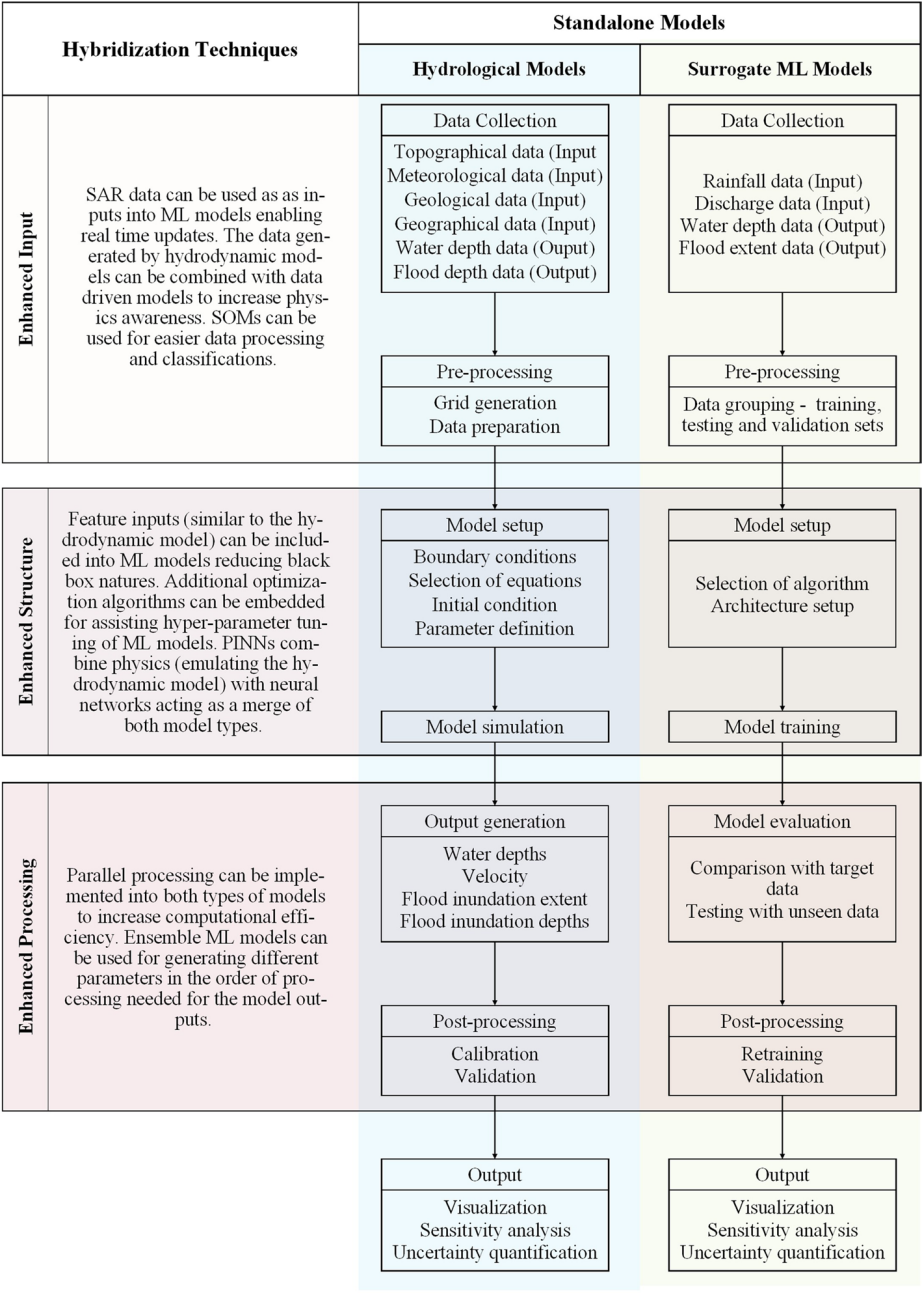
Structure of standalone hydrodynamic and machine learning models and areas of hybridization applied

Standalone hydrodynamic models and ML models used as surrogates have different schematics as shown in Fig. 4, however, the structures can be grouped parallel to each other. Enhancing the inputs can be done to the data collection and pre-processing phases, the structure can be enhanced at the model setup and simulation/training phases, and the processing can be enhanced at the output generation/model evaluation and the post-processing phases.

### Diversity of assessment metrics

Review of the studies indicate numerous metrics used (supplementary data Table 7) in evaluating models and broad grouping allowed classification of the metrics into categories of accuracy-based metrics, and speed-based metrics (Table 2). Metrics are often used simultaneously with a prevalence from the accuracy category.

**Table 2:** Categorized metrics used for the evaluation of hybrid flood models

Category	Sub-category	Metrics	Description
Accuracy based	Absolute errors	Mean Absolute Error (MAE)	Measures the average magnitude of the deviation between simulated and observed values, without emphasizing large errors
		Mean Absolute Deviation (MAD)	
		Mean absolute percentage error (MAPE)	
	Squared error	Mean Square Error (MSE)	Penalizes larger deviations more strongly by squaring the error; useful for evaluating models sensitive to extreme inaccuracies
		Sum of Squared Error (SSE)	
		Root Mean Square Error (RMSE)	
		Relative Root Mean Square Error (RRMSE)	
	Event specific	Maximum Error (MaxE)	Assesses model performance at critical points of a flood event
		Error to peak time (ETP)	
	Continuous accuracy	Nash-suttcliffe efficiency (NSE)	Evaluates overall agreement between simulated and observed continuous variables
	Classification and detection	Overall Accuracy (OA)	Indicates the overall proportion of correct simulations across all categories
		Producer Accuracy (PA)	
		User Accuracy (UA)	
		Classification Accuracy rate (CA)	
		Reciver Operating Characteristic Curve (ROC)	
		Precision	
		Recall	
		Probability Of Detection (POD)	
		False Alarm Ratio (FAR)	
		Sucess Ratio (SR)	
		Threat Score (TS)	
		F1 Score	
	Goodness of fit	Coefficient of determination (R$^2$)	Statistical or similarity tests to compare reference and simulated data distributions
		Pearson Correlation Coefficient (PCC)	
		Wilcoxon Signed Rank Test	
		Friedman Test	
		Structural Similarity Index (SSIM)	
		Kappa Score	
	Bias	Bias	Measures the tendency of the model to over/underestimate values
		Normalized Bias	
Speed based		Speed Up	Assesses computational efficiency
		Megacells Per Second (MPS)	
		I/O Seconds	

Accuracy-based metrics reflect on how well the model simulations/predictions match the reference data, where different metrics account for the errors, outliers, correct and incorrect simulations. They can also reflect on patterns, distributions and error behaviors, and they can account for performance consistency, variance, and relative accuracy of simulations. Speed-based metrics indicate the timeliness of model processing and is useful in determining effectiveness on real-time applications. Despite the existence of numerous metrics for model evaluation, similar metrics are often used in combination, usually from the accuracy class. This can result in scores that are repetitive on the errors analyzed. The use of interchanging metrics and scores result the inability to compare models during selection processes. Despite the use of many common performance metrics in flood model analysis (Table 2), the scores depend on the structure of the model, the application, and the method of analysis. For instance, as the models become more complex, vary in flood characteristics, and have higher resolution, the values of calculated metrics would be different from the same metrics calculated for a model in a simple domain. It is highlighted that a standard unified framework is required to benchmark models, and that a formal method of selecting suitable hybrid models with appropriate data availability and model complexity is needed. The use of metrics from each main class and distributed across the sub-categories would be helpful in ensuring that the metrics presented are not too similar and would cover the extent of the evaluation of the characteristics of the novel model. In turn, the scores would also be indicative of the desirable aspects of a hybrid model.

## Discussion

### Do hybrid models have advantages over standalone models?

With increasing urgency for effective flood management, there is a surge in the development of numerical, statistical and ML models for flood forecasting and nowcasting. Commonly used flood models are usually standalone (single) numerical models that follow the laws of hydrodynamics and solve the shallow water equations (Zanchetta and Coulibaly 2022). However, standalone numerical models have prominent tradeoffs between accuracy and resolution, and highly accurate models with high resolutions are computationally expensive and are not suited for real-time simulations (short-term applications). They are used to simulate a variety of diverse flooding scenarios but can be limited by their requirement of computational power, lack of short-term application, and the need of high-resolution temporal and spatial hydro-geomorphological data (Mosavi et al. 2018). The schematic of a process-based model involves the input data (usually observed) used to build the model, processing based on hydrodynamic principles, testing, calibration and validation before application (Fig. 4). The relationships derived are fully based on the laws of physics and the calculations are transparent, however, depending on the extent of the catchment, the resolution and necessary accuracy, and the nature of the input data, the time and effort spent on building, running and calibrating a model can vary largely. This highlights the tradeoffs and call for methods that make these limitations less prominent. ML models commonly used to emulate hydrodynamic models can be faster and more computationally efficient, however, they are blackbox models that map patterns between inputs and outputs. Hybridization aims to improve standalone models for more efficient flood inundation modeling. With an increasing trend of research on hybrid flood models, in this section we discuss the necessity of these models in comparison to standalone models.

Reviewed literature demonstrates many advantages of hybrid models. Several studies have noted their rapid prediction ability, with real time applications (Schmid and Leandro 2023; Tewari et al. 2021; Pan et al. 2011) indicating that hybrid models can be useful in hazard mitigation. A main goal of hybridization is faster prediction compared to conventional approaches, and studies have demonstrated speed-ups (García-Feal et al. 2018; Dang et al. 2024; Fraehr et al. 2023), compared to numerical and commercial models (Liao et al. 2023; Guidolin et al. 2016; Wijaya and Yang 2018). Accuracy is also an important metric in flood prediction, and studies by Chen et al. (2022); Adriano et al. (2023); Kao et al. (2021); Panahi et al. (2022); Feng et al. (2023) have stated good/acceptable accuracy in predictions using hybrid models. They were also noted to outperform single ML models (Tien Bui et al. 2019; Ngo et al. 2021; Ebtehaj and Bonakdari 2022 ) with more similarities with numerical model predictions (Wijaya and Yang 2021). Another advantage is their applicability in data-scarce areas (Sirsant et al. 2024; Xie et al. 2021; Tripathi and Mohanty 2024), in comparison to numerical and ML models which are data intensive. Hybrid models also demonstrated lower computational cost (Li and Willems 2020; Xu and Gao 2024; Lee et al. 2024), with reduced CPU times (Chang et al. 2010). Mosavi et al. (2018) notes hybridization to be one of the most effective strategies in improving single ML flood models, while Bentivoglio et al. (2022) notes that hybrid models are faster than numerical methods and can have high accuracy in predictions.

Despite the promising research on hybrid models and their strengths noted in literature, there are still limitations of these models that need further improvement. They still remain quite data-dependent due to their ML model base, and accuracy depends on the input data used to train these models (Li and Willems 2020; Xie et al. 2021). Bentivoglio et al. (2022) also notes difficulty in generalizing to unseen cases, and historical records and survey data may be too sparse to train a model (Fang et al. 2022). There is also a need for specialized expertise in building and using these models (Marshall et al. 2017). Another point of focus is their lack of physics (except physics informed ML models) followed in mapping relationships, in comparison to hydrodynamic models which are built on equations. Eventually, the usefulness and applicability of a hybrid model depend on the prioritized characteristics for a specific application. For example, if speed is valued for real time operations, a hybrid model may be more advantageous than a hydrodynamic model, and may be more accurate than a standalone ML model. Consequently, if accuracy is valued more, an existing calibrated hydrodynamic model may be preferred for long term predictions. This again highlights the importance in benchmarking which gives the ability of comparing and evaluating models objectively.

###  Benchmarking framework

Evaluation metrics can be used to determine the relative success of the model, however, a standardized framework of benchmarking is needed to compare and select models for different applications. This was done for physics based models in the past decade, and hybrid models should follow suit. A benchmarking method that incorporates standard metrics and standardized datasets would enable the comparison of models for better applicability in different domains. For this we refer to Hunter et al. (2008) where the authors compare 2D hydraulic models in terms of relative performance and response to topographic error, and conduct a sensitivity analysis to determine if the differences in models can be matched by calibration using realistic parameters. We also refer to Neelz and Pender (2010) for structured benchmarking where a series of tests are proposed for hydraulic packages.

In the context of hybrid models, it is crucial to benchmark and compare the novel model to existing hydrodynamic models and standalone ML models to determine if hybridization is more advantageous and where the most beneficial application lies. We propose a framework of benchmarking which starts by classifying the model based on the method of hybridization used, and quantitatively by the catchment scale and resolution. This first step builds up to the selection of the case studies and standard data required to test and evaluate the model. Other factors to be considered in evaluation is the application required of the model and the target prediction variables. Table 3 shows the classifications that are suggested.

**Table 3:** Classification of model type for case studies

Category	Option 1	Option 2	Option 3
Hybridization (A)	Enhanced input	Enhanced structure	Enhanced processing
Catchment scale (B)	Small(< 100 km^2^)	Medium (100 – 1000 km^2^)	Large (> 1000 km^2^)
Resolution (C)	Fine (< 10 m)	Medium (10 – 50 m)	Coarse (> 50 m)
Target data (D)	Flood inundation	Flood susceptibility	Flood hazard
Application (E)	Nowcasting	Forecasting	Hindcasting

Hypothetical case studies can be used to test these models using standard data, where the objective is to compare the hybrid model, a hydrodynamic model, and a standalone ML model (emulating the hydrodynamic model) in the same conditions to determine the relative performance. Therefore, a single case study (which we later group further) should include a calibrated hydrodynamic model, a standalone ML model, catchment details and inputs (eg: DEM data, channel data, roughness, SAR data), boundary conditions (eg: hydrograph data, river flow data, rainfall), target outputs flood variables/maps (eg: depth, extent, probability, risk, return-period), and model evaluation data (unseen boundary conditions and target outputs). In comparing the models, evaluation/performance metrics can be used, and the same computational resources should be used to train and run all the models. The data used for model validation and testing should be completely unseen in the earlier steps to ensure unbiased processing. When building the case studies, we propose multiple combinations/groups of openly available standardized datasets. For example, grouping the data by catchment scale would yield multiple sub-groups of small catchment scale (with fine, medium and coarse resolutions), and similarly for the medium and large catchment scales. This alone would present nine case studies that can be selected based on the hybrid model structure.

**Fig. 5:**
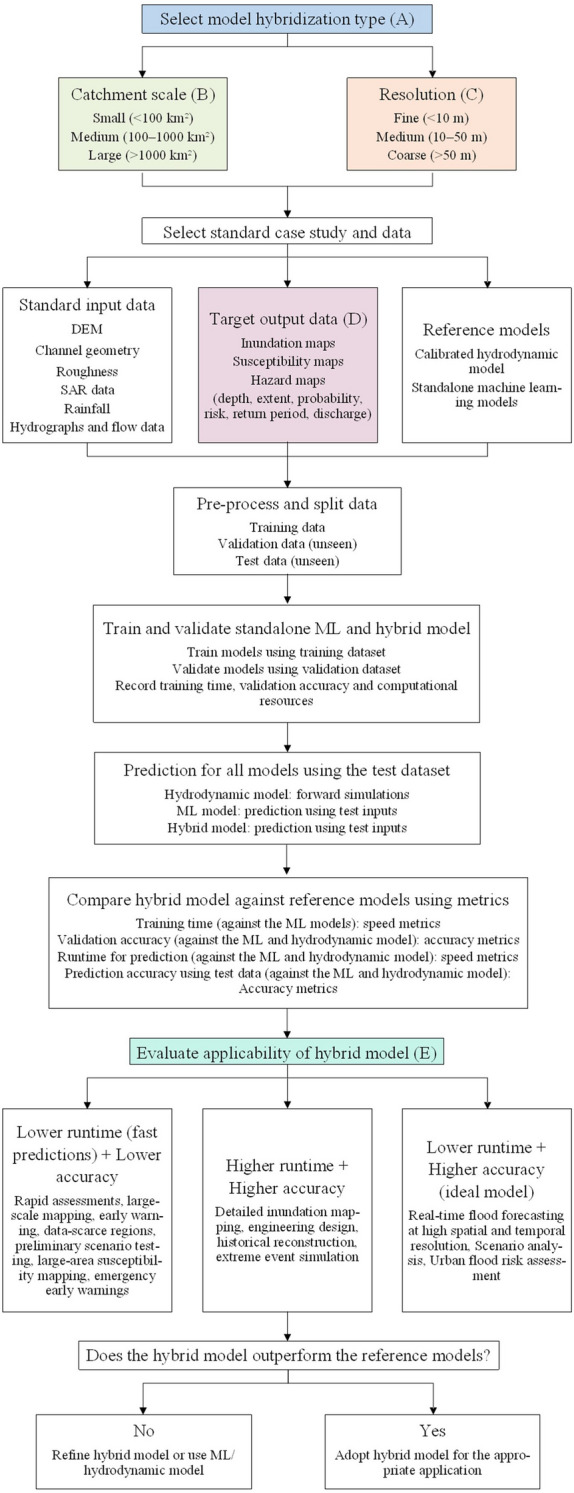
Benchmarking flowchart for selection of models and case studies

We also propose standard tests to compare the model with the relevant categories of performance metrics. Model training time can be evaluated between the hybrid and standalone ML model using speed based metrics. For all three models, validation can be tested using accuracy based metrics, prediction runtime can be tested using speed metrics, and prediction accuracy using unseen data can be evaluated using accuracy metrics. Based on these comparisons, the overall runtimes and accuracies of the models can be compared to determine the applications the hybrid model is suited for. If the models are not benchmarked using standard data, a separate hydrodynamic model and standalone ML model must be built for the same catchment/dataset for comparison. This would help evaluate the hybrid model following the same structure given in the tests. Figure 5 showcases the framework proposed.

Furthermore, we propose the division of evaluation metrics into primary and secondary categories to ensure easier comparison between models. Primary metrics are the minimal set of metrics that should be used to evaluate the model. These metrics are widely used, interpretable and capture the core model performance aspects such as magnitude of errors, accuracy, flood detection ability, goodness of fit and computational efficiency. Using these metrics in benchmarking scores would allow cross-model comparison. Secondary metrics are optional and can be context specific. They can be used as complementary scores to further evaluate the model and detailed analysis, but would not be necessary for standard benchmarking. Table 4 presents the hierarchy of primary and secondary metrics.

**Table 4:** Consolidated hierarchy of metrics used for hybrid flood model evaluation. Primary metrics are recommended for benchmarking, while secondary metrics are diagnostic or optional

Metric type	Primary metric(s)	Secondary metrics
Absolute error	MAE	MAD, MAPE
Squared error	RMSE	MSE, SSE, RRMSE
Event-specific error	MaxE	ETP
Accuracy (continuous)	NSE	–
Classification / detection	Precision, Recall, F1 or TS	CA, OA, UA, PA, POD, FAR, SR, ROC
Goodness-of-fit & statistical comparison	R²	PCC, SSIM, Kappa, Wilcoxon test, Friedman test
Bias	Bias	Normalized Bias
Speed	Speed-up	MPS, I/O Seconds

As an example, we refer to the physics-informed ML model presented by Donnelly et al. (2024) which acts as a surrogate for numerical hydrodynamic simulators (LISFLOOD-FP and Delft3D) in flood modeling.. A CNN architecture was used as the base of the neural network, and the evaluation metrics used were RMSE and mRMSE (modified RMSE). Synthetic input data was used to simulate inundation resulting from river overflow during low-probability, high-impact, short-term flood events in Tadcaster, UK (approximately 5 km^2^). A single CNN model was noted to converge faster during training, however, the PINN model reached a more stable and lower minimum validation RMSE, and showed an improved mRMSE with lower volume residuals indicating a more physically consistent performance. The study reports comparisons between the hybrid ML model (PINN) and the standalone CNN model, and is therefore benchmarked to an extent. Applying the framework suggested would enable the comparison of the ML models and the hydrodynamic model as well, and would clearly show the advantages and limitations of using a hybrid model. These models fall under; (A) Enhanced structure, (B) Small catchment scale, (C) Fine resolution. Using a standard case study matching these categories would enable progressing through the flowchart and benchmarking the hybrid PINN model more comparatively and thoroughly. Standard inputs, target outputs would be provided and could be used to train the PINN and CNN model, validate and test against each other and the calibrated hydrodynamic model.

### A way forward in PINNs

Eventually, complex hydrodynamics drive the emerging frontrunners of hybrid models and hybridization techniques used. The most prominent compromise of standalone ML models that are used as surrogates is between the speed of the model and the black-box nature. The lack of physics highlighted throughout this research is a leading factor in needing hybridization of ML models, and hybridization is shown to be beneficial in reducing the tradeoffs between accuracy and speed. Physics informed neural networks (PINNs) introduce physics loss functions into the base ML architecture, where the model is penalized for violating physics laws. This increases the physical awareness of the model, and the combined data and physics losses are balanced using a weight factor. The focus on PINNs is increasing, however, currently the research on PINNs applied to flood modeling remain rather scarce. The studies surrounding PINNs in the reviewed literature have demonstrated the strengths of PINNs. Donnelly et al. (2023) developed a surrogate PINN to emulate a hydrodynamic simulator, and noted that the PINN model outperformed a single CNN surrogate across multiple metrics (10 - 20 percent). Despite the reduced volume discrepancy and better robustness in predictions, the PINN required increased effort and computational resouces. A geo-PINN presented by Xu et al. (2024) could accurately predict flood extents and depths (MAE of 0.061 m), and outperformed traditional hydrodynamics in spatial detail. The PINN was reported to be computationally efficient for large-scale simulations and real time flood hazard forecasting, however, limitations were noted in the dependancy on observed data and introduction of errors due to downscaling temporal resolution. A PINN proposed by Donnelly et al. (2024) was aimed at predicting spatiotemporal variations in water surface elevations, and reportedly outperformed (RMSE) data-driven CNN surrogates by  25 percent for LISFLOOD-FP and  11 percent for Delft3D, with volume discrepancy reduced by  20 percent. Models were noted to show stable optimization behavior, high data efficiency, and robustness across boundary conditions.

PINNs are an upcoming avenue (all works reviewed were very recent) for building physics-informed ML models that can be more generalizable and suited for real-time applicability. Used conjointly with the other methods of hybridization categorized in this research, PINN models can be improved further. For example, combining a PINN model can be combined with SAR data for rapid updates of input data, targeted optimization could be done for more effective hyper-parameter tuning, and parallel computing could be incorporated for distributing the processes for higher computational efficiency. However, PINNs tend to be more complex than simple surrogate ML models, and would be overcomplicated for applications that do not require high accuracy and are applied to simple domains. In this scenario, feature-informed ensemble models could be an option where SAR data can be incorporated for real-time processing. Similarly, existing hydrodynamic models could be used to generate inputs to ML models for reasonable mapping of non-linear inputs and outputs. Eventually, the model architectures used and the hybridization techniques depend on the application needed from the model. PINNs are shown to have a distinctive edge over standalone models as evidenced by the literature reviewed. With further research, PINNs could be a way forward in hybrid flood modeling.

## Conclusions

Hybridization of flood inundation models aim to reduce the trade-offs between accuracy, resolution, and computational efficiency that is prominent in standalone models. In this research, we explored the common ML methods used in building flood inundation models, categorized the methods of hybridization and the performance analysis metrics. Although the frontrunners of ML methods indicated a good capacity to act as surrogate models, their black-box natures and lack of physics act as deterrents for use as effective flood models. We explored the different ideas of hybridization presented, and proposed that hybridization can be defined broadly as the combination of the strengths of standalone process-based and ML models to enhance the input, structure or processing of the hybrid model. We discussed the advantages of using hybrid models in comparison to standalone hydrodynamic and ML models, and noted the reported strengthens and limitations. With the vast variation of new models that can be built by combining these methods, benchmarking these models become crucial, and the use of performance metrics in comparing models should be done carefully. We suggested a standardized framework that quantitatively groups models based on the hybridization technique used, catchment scale and resolution, and proposed a series of tests to evaluate the hybrid model against their single counterparts using case studies. We also classified performance metrics into primary and secondary scores that would help determine the usefulness of the hybrid model for it’s intended application. With the increasing trend in research on hybrid flood models, we explore a way forward in using PINNs that combine physics laws with ML techniques, further improving the models realism and robustness.

## Supplementary Information

Below is the link to the electronic supplementary material.

Supplementary file 1 (pdf 228 KB)

## References

[CR1] Adriano B, Yokoya N, Yamanoi K, Oishi S (2023) Combining deep learning and numerical simulation to predict flood inundation depth. In: International Geoscience and Remote Sensing Symposium (IGARSS), Institute of Electrical and Electronics Engineers Inc.. pp 1154–1157. 10.1109/IGARSS52108.2023.10282463

[CR2] Balakrishna Madayala A, Jain A, Lohani B (2022) Development of a physics-guided neural network model for effective Urban flood management. J Hydrol Eng. 10.1061/(asce)he.1943-5584.0002196

[CR3] Beevers L, Collet L, Aitken G, Maravat C, Visser A (2020) The influence of climate model uncertainty on fluvial flood hazard estimation. Nat Hazards 104:2489–2510. 10.1007/s11069-020-04282-4

[CR4] Bentivoglio R, Isufi E, Jonkman SN, Taormina R (2022) Deep learning methods for flood mapping a review of existing applications and future research directions. Hydrol Earth Syst Sci Discuss. 10.5194/hess-26-4345-2022

[CR5] Chang LC, Amin MZM, Yang SN, Chang FJ (2018) Building ANN-based regional multi-step-ahead flood inundation forecast models. Water (Switzerland). 10.3390/W10091283

[CR6] Chang LC, Shen HY, Chang FJ (2014) Regional flood inundation nowcast using hybrid SOM and dynamic neural networks. J Hydrol 519:476–489. 10.1016/j.jhydrol.2014.07.036

[CR7] Chang LC, Shen HY, Wang YF, Huang JY, Lin YT (2010) Clustering-based hybrid inundation model for forecasting flood inundation depths. J Hydrol 385:257–268. 10.1016/j.jhydrol.2010.02.028

[CR8] Chen M, Li Z, Gao S, Xue M, Gourley JJ, Kolar RL, Hong Y (2022) A flood predictability study for Hurricane Harvey with the CREST-iMAP model using high-resolution quantitative precipitation forecasts and U-Net deep learning precipitation nowcasts. J Hydrol. 10.1016/j.jhydrol.2022.128168

[CR9] Dang TQ, Tran BH, Le QN, Dang TD, Tanim AH, Pham QB, Bui VH, Mai ST, Thanh PN, Anh DT (2024) Application of machine learning-based surrogate models for urban flood depth modeling in Ho Chi Minh City, Vietnam [Formula presented]. Appl Soft Comput. 10.1016/j.asoc.2023.111031

[CR10] Darabi H, Rahmati O, Naghibi SA, Mohammadi F, Ahmadisharaf E, Kalantari Z, Torabi Haghighi A, Soleimanpour SM, Tiefenbacher JP, Tien Bui D (2022) Development of a novel hybrid multi-boosting neural network model for spatial prediction of urban flood. Geocarto Int 37:5716–5741. 10.1080/10106049.2021.1920629

[CR11] Donnelly J, Daneshkhah A, Abolfathi S (2023) A physics-informed neural network surrogate model for tidal simulations. In: UNCECOMP Proceedings, National Technical University of Athens. 10.7712/120223.10379.19908

[CR12] Donnelly J, Daneshkhah A, Abolfathi S (2024) Physics-informed neural networks as surrogate models of hydrodynamic simulators. Sci Total Environ. 10.1016/j.scitotenv.2023.168814 10.1016/j.scitotenv.2023.168814 38016570

[CR13] Ebtehaj I, Bonakdari H (2022) A reliable hybrid outlier robust non-tuned rapid machine learning model for multi-step ahead flood forecasting in Quebec. Canada J Hydrol. 10.1016/j.jhydrol.2022.128592

[CR14] Fang L, Huang J, Cai J, Nitivattananon V (2022) Hybrid approach for flood susceptibility assessment in a flood-prone mountainous catchment in China. J Hydrol. 10.1016/j.jhydrol.2022.128091

[CR15] Fares A, Aljohani H, Alkhodre AB, Ahamad A, Sen A, Ramazan MS, Alzahrani B, Siddiqui MS (2023) Flood prediction using hydrologic and ML-based modeling: a systematic review. Tech Rep 11. www.ijacsa.thesai.org

[CR16] Feng D, Tan Z, He QZ (2023) Physics-informed neural networks of the Saint-Venant equations for downscaling a large-scale river model. Water Resour Res. 10.1029/2022WR033168

[CR17] Fraehr N, Wang QJ, Wu W, Nathan R (2022) Upskilling low-fidelity hydrodynamic models of flood inundation through spatial analysis and Gaussian process learning. Water Resour Res. 10.1029/2022WR032248

[CR18] Fraehr N, Wang QJ, Wu W, Nathan R (2023) Development of a fast and accurate hybrid model for floodplain inundation simulations. Water Resour Res. 10.1029/2022wr033836

[CR19] French J, Mawdsley R, Fujiyama T, Achuthan K (2017) Combining machine learning with computational hydrodynamics for prediction of tidal surge inundation at estuarine ports. In: Procedia IUTAM, Elsevier B.V.. pp 28–35. 10.1016/j.piutam.2017.09.005

[CR20] Gao W, Liao Y, Chen Y, Lai C, He S, Wang Z (2024) Enhancing transparency in data-driven urban pluvial flood prediction using an explainable CNN model. J Hydrol. 10.1016/j.jhydrol.2024.132228

[CR21] García-Feal O, González-Cao J, Gómez-Gesteira M, Cea L, Domínguez JM, Formella A (2018) An accelerated tool for flood modelling based on Iber. Water (Switzerland). 10.3390/w10101459

[CR22] Guidolin M, Chen AS, Ghimire B, Keedwell EC, Djordjević S, Savić DA (2016) A weighted cellular automata 2D inundation model for rapid flood analysis. Environ Model Softw 84:378–394. 10.1016/j.envsoft.2016.07.008

[CR23] Habibi A, Delavar MR, Nazari B, Pirasteh S, Sadeghian MS (2023) A novel approach for flood hazard assessment using hybridized ensemble models and feature selection algorithms. Int J Appl Earth Obs Geoinf 122:103443. 10.1016/j.jag.2023.103443

[CR24] Hinge G, Sirsant S, Kumar A, Gupta R, Hamouda MA (2024) Enhancing flood prediction in Southern West Bengal, India using ensemble machine learning models optimized with symbiotic organisms search algorithm. Stochastic Environ Res Risk Assess 10.1007/s00477-024-02712-4

[CR25] Hunter NM, Bates PD, Neelz S, Pender G, Villanueva I, Wright NG, Liang D, Falconer RA, Lin B, Waller S, Crossley AJ, Mason DC (2008) Benchmarking 2D hydraulic models for urban flooding. Proc Inst Civ Eng Water Manag 161:13–30. 10.1680/wama.2008.161.1.13

[CR26] Jeba GS, Chitra P (2024) Flood prediction through hydrological modeling of rainfall using Conv1D-SBiGRU algorithm and RDI estimation: a hybrid approach. Stoch Env Res Risk Assess 38:3587–3606. 10.1007/s00477-024-02768-2

[CR27] Jhong BC, Lin CY, Jhong YD, Chang HK, Chu JL, Fang HT (2022) Assessing the effective spatial characteristics of input features through physics-informed machine learning models in inundation forecasting during typhoons. Hydrol Sci J 67:1527–1545. 10.1080/02626667.2022.2092406

[CR28] Jhong YD, Chen CS, Lin HP, Chen ST (2018) Physical hybrid neural network model to forecast typhoon floods. Water (Switzerland). 10.3390/w10050632

[CR29] Kabir S, Patidar S, Pender G (2021) A machine learning approach for forecasting and Visualising flood inundation information. Proc Inst Civ Eng Water Manag 174:27–41. 10.1680/jwama.20.00002

[CR30] Kabir S, Patidar S, Xia X, Liang Q, Neal J, Pender G (2020) A deep convolutional neural network model for rapid prediction of fluvial flood inundation. J Hydrol. 10.1016/j.jhydrol.2020.125481

[CR31] Kao IF, Liou JY, Lee MH, Chang FJ (2021) Fusing stacked autoencoder and long short-term memory for regional multistep-ahead flood inundation forecasts. J Hydrol. 10.1016/j.jhydrol.2021.126371

[CR32] Kurian C, Sudheer KP, Vema VK, Sahoo D (2020) Effective flood forecasting at higher lead times through hybrid modelling framework. J Hydrol. 10.1016/j.jhydrol.2020.124945

[CR33] Kurz S, De Gersem H, Galetzka A, Klaedtke A, Liebsch M, Loukrezis D, Russenschuck S, Schmidt M (2022) Hybrid modeling: towards the next level of scientific computing in engineering. J Math Ind. 10.1186/s13362-022-00123-0

[CR34] Lee CC, Huang L, Antolini F, Garcia M, Juan A, Brody SD, Mostafavi A (2024) Predicting peak inundation depths with a physics informed machine learning model. Sci Rep. 10.1038/s41598-024-65570-8 10.1038/s41598-024-65570-8 PMC11211320 38937603

[CR35] Li X, Willems P (2020) A hybrid model for fast and probabilistic Urban pluvial flood prediction. Water Resour Res. 10.1029/2019WR025128

[CR36] Liao Y, Wang Z, Lai C, Xu CY (2023) A framework on fast mapping of Urban flood based on a multi-objective random forest model. Int J Disaster Risk Sci 14:253–268. 10.1007/s13753-023-00481-2

[CR37] Liu B, Li X, Zheng G (2019) Coastal inundation mapping from bitemporal and dual-polarization SAR imagery based on deep convolutional neural networks. J Geophys Res Oceans 124:9101–9113. 10.1029/2019JC015577

[CR38] Marshall R, Ghafoor SK, Kalyanapu AJ, Rogers M, Dullo TT (2017) Performance improvement of a two-dimensional flood simulation application in hybrid computing environments. In: Proceedings - 2017 5th International Symposium on Computing and Networking, CANDAR 2017, Institute of Electrical and Electronics Engineers Inc.. pp 21–29. 10.1109/CANDAR.2017.106

[CR39] Mosavi A, Ozturk P, Chau KW (2018) Flood prediction using machine learning models literature review. Water. 10.3390/w10111536

[CR40] Munawar HS, Hammad AW, Waller ST (2021) A review on flood management technologies related to image processing and machine learning. Automat Construct. 10.1016/j.autcon.2021.103916

[CR41] Neelz S, Pender G (2010) Benchmarking of 2D hydraulic modelling packages. Environment Agency

[CR42] Ngo PTT, Hoang ND, Pradhan B, Nguyen QK, Tran XT, Nguyen QM, Nguyen VN, Samui P, Bui DT (2018) A novel hybrid swarm optimized multilayer neural network for spatial prediction of flash floods in tropical areas using sentinel-1 SAR imagery and geospatial data. Sensors (Switzerland). 10.3390/s18113704 10.3390/s18113704 PMC6263740 30384451

[CR43] Ngo PTT, Pham TD, Nhu VH, Le TT, Tran DA, Phan DC, Hoa PV, Amaro-Mellado JL, Bui DT (2021) A novel hybrid quantum-PSO and credal decision tree ensemble for tropical cyclone induced flash flood susceptibility mapping with geospatial data. J Hydrol. 10.1016/j.jhydrol.2020.125682

[CR44] Oliveira A, de Jesus G, Rogeiro J, Fernandes J, Rodrigues R (2022) An hybrid methodology for integrated flood forecasting from the watershed to the sea. In: Proceedings of the IAHR World Congress, International Association for Hydro-Environment Engineering and Research. pp 4941–4946. 10.3850/IAHR-39WC2521716X2022737

[CR45] Pan TY, Lai JS, Chang TJ, Chang HK, Chang KC, Tan YC (2011) Hybrid neural networks in rainfall-inundation forecasting based on a synthetic potential inundation database. Nat Hazards Earth Syst Sci 11:771–787. 10.5194/nhess-11-771-2011

[CR46] Panahi M, Rahmati O, Kalantari Z, Darabi H, Rezaie F, Moghaddam DD, Ferreira CSS, Foody G, Aliramaee R, Bateni SM, Lee CW, Lee S (2022) Large-scale dynamic flood monitoring in an arid-zone floodplain using SAR data and hybrid machine-learning models. J Hydrol. 10.1016/j.jhydrol.2022.128001

[CR47] Rahmati O, Darabi H, Panahi M, Kalantari Z, Naghibi SA, Ferreira CSS, Kornejady A, Karimidastenaei Z, Mohammadi F, Stefanidis S, Tien Bui D, Haghighi AT (2020) Development of novel hybridized models for urban flood susceptibility mapping. Sci Rep. 10.1038/s41598-020-69703-7 10.1038/s41598-020-69703-7 PMC7395144 32737384

[CR48] Rathnasiri P, Adeniyi O, Thurairajah N (2023). Data-driven approaches to built environment flood resilience a scientometric and critical review. 10.1016/j.aei.2023.102085

[CR49] Sarwar J, Khan SA, Azmat M, Khan F (2024) An application of hybrid bagging-boosting decision trees ensemble model for riverine flood susceptibility mapping and regional risk delineation. Water Resour Manag. 10.1007/s11269-024-03995-6

[CR50] Schmid F, Leandro J (2023) A feature-informed data-driven approach for predicting maximum flood inundation extends. Geosciences (Switzerland). 10.3390/geosciences13120384

[CR51] Sharma NK, Saharia M (2025) DeepSARFlood: rapid and automated SAR-based flood inundation mapping using vision transformer-based deep ensembles with uncertainty estimates. Sci Remote Sens. 10.1016/j.srs.2025.100203

[CR52] Sirsant S, Hinge G, Singh H, Hamouda MA (2024) A hybrid convolutional neural network model coupled with AdaBoost regressor for flood mapping using geotagged flood photographs. Nat Hazards. 10.1007/s11069-024-07041-x

[CR53] Situ Z, Zhong Q, Zhang J, Teng S, Ge X, Zhou Q, Zhao Z (2025) Attention-based deep learning framework for urban flood damage and risk assessment with improved flood prediction and land use segmentation. Int J Disaster Risk Reduct. 10.1016/j.ijdrr.2024.105165

[CR54] Sun AY, Li Z, Lee W, Huang Q, Scanlon BR, Dawson C (2023) Rapid flood inundation forecast using fourier neural operator. Tech Rep

[CR55] Tewari A, Kshemkalyani V, Kukreja H, Menon P, Thomas R, (2021) Application of LSTMs and HAND in rapid flood inundation mapping. In: Proceedings - 5th International Conference on Intelligent Computing and Control Systems, ICICCS 2021, Institute of Electrical and Electronics Engineers Inc.. pp 515–521. 10.1109/ICICCS51141.2021.9432332

[CR56] Tien Bui D, Hoang ND, Pham TD, Ngo PTT, Hoa PV, Minh NQ, Tran XT, Samui P (2019) A new intelligence approach based on GIS-based multivariate adaptive regression splines and metaheuristic optimization for predicting flash flood susceptible areas at high-frequency tropical typhoon area. J Hydrol 575:314–326. 10.1016/j.jhydrol.2019.05.046

[CR57] Tripathi V, Mohanty MP (2024) Can geomorphic flood descriptors coupled with machine learning models enhance in quantifying flood risks over data-scarce catchments? Environmental Science and Pollution Research, Development of a hybrid framework for Ganga basin (India). 10.1007/s11356-024-33507-3 10.1007/s11356-024-33507-3 38709408

[CR58] Wang Z, Lyu H, Fu G, Zhang C (2024) Time-guided convolutional neural networks for spatiotemporal urban flood modelling. J Hydrol. 10.1016/j.jhydrol.2024.132250

[CR59] Wijaya OT, Yang TH (2021) A novel hybrid approach based on cellular automata and a digital elevation model for rapid flood assessment. Water (Switzerland). 10.3390/w13091311

[CR60] Wijaya OT, Yang TH, (2024) Application of parallel computing on hybrid inundation model, case study: chiayi county flood 23-24 August 2018. In: IOP Conference Series: Earth and Environmental Science, Institute of Physics. 10.1088/1755-1315/1343/1/012017

[CR61] Xie S, Wu W, Mooser S, Wang Q, Nathan R, Huang Y (2021) Artificial neural network based hybrid modeling approach for flood inundation modeling. J Hydrol 592: 125605. https://linkinghub.elsevier.com/retrieve/pii/S0022169420310660, 10.1016/j.jhydrol.2020.125605

[CR62] Xu K, Han Z, Xu H, Bin L (2023) Rapid prediction model for Urban floods based on a light gradient boosting machine approach and hydrological-hydraulic model. Intl J Disaster Risk Sci 14:79–97. 10.1007/s13753-023-00465-2

[CR63] Xu L, Gao L (2024) A hybrid surrogate model for real-time coastal urban flood prediction: an application to Macao. J Hydrol. 10.1016/j.jhydrol.2024.131863

[CR64] Xu Q, Shi Y, Bamber JL, Ouyang C, Zhu XX (2024) Large-scale flood modeling and forecasting with FloodCast. Water Res. 10.1016/j.watres.2024.122162 10.1016/j.watres.2024.122162 39126745

[CR65] Yang TH, Hwang GD, Tsai CC, Ho JY (2016) Using rainfall thresholds and ensemble precipitation forecasts to issue and improve urban inundation alerts. Hydrol Earth Syst Sci 20:4731–4745. 10.5194/hess-20-4731-2016

[CR66] Zanchetta AD, Coulibaly P (2022) Hybrid surrogate model for timely prediction of flash flood inundation maps caused by rapid river overflow. Forecasting 4:126–148. 10.3390/forecast4010007

