# Supplementary material for: Hybrid methods in flood inundation modeling: a systematic review
Source: Nat Hazards (Dordr). 2026 Apr 2;122(8):357. doi: 10.1007/s11069-026-08078-w (PMC13046610; doi:10.1007/s11069-026-08078-w)
Supplement: Supplementary file 1 — (pdf 228 KB) [file 11069_2026_8078_MOESM1_ESM.pdf]

7. Appendix

7.1. State of the art hybrid architectures used in flood inundation models

Table 5 further describes the classified techniques through which different data-driven models can be hybridized. Different hybridization methods have specific advantages and limitations, and often multiple methods are combined to enhance the strengths and reduce the drawbacks. The analysis of the models was done by comparing the novel hybrid models to the reference data used, and the consensus is that hybridizing improves performance and speed. However, improvements are also suggested for these models to reduce the impact of their limitations.

Type	Description	Advantages and Disadvantages	Future directions

Table 5: Methods of hybridization used in hybrid flood inundation models

**Type**	**Description**	**Advantages and Disadvantages**	**Future directions**
SAR integrated data-driven models (enhanced input / training)	SAR generates a vast amount of high resolution data which is spatially well distributed and the generated datasets or inundation maps can be used to train the ML models. The ML models can be used for flood detection, flood hazard mapping, land monitoring etc.	• Can be operated under any condition (sunlight and cloud cover) to give reliable near real time information<br>• Can provide rapid information with high resolution, wider coverage, lower cost and good continuity for monitoring dynamic events<br>• Can detect water presence, soil saturation, and surface changes<br>• SAR data is sometimes measured only at large time intervals and can have speckle noise and back-scatter that can reduce image quality and impact the ML model<br>• ML models built on SAR can be data dependent and has no physical constraints implied<br>• Data needs pre-processing to be used for training, and an ML model trained on one sensor may not work well with others	DL models with deep feature extraction can be used, and incorporated with other data like optical images, DEMs and rainfall data (multi-modal data). Self organizing maps (SOMs) that are unsupervised learning neural networks that can be used for clustering and providing visual insights in high definition datasets. Learning of the ML model can be enhanced by application to a range of study areas.

Type	Description	Advantages and Disadvantages	Future directions
Data-driven models trained by numerical models (enhanced input / training)	Numerical models (hydrological, hydraulic or hydrodynamic) can be used to build datasets to train the ML models. For case-studies, observed input data such as rainfall and DEMs are used alongside other parameters to calibrate the numerical models to match observed data of water depth, inundation extent etc. Synthetic data can also be generated and the output inundation maps can be used to train the ML models which act as surrogates.	• The ML models are trained on physically based inputs and outputs, with increased awareness of constraints.<br>• Abundant data can be generated including rare and extreme events for training the models.<br>• Widely used in flood modeling<br>• Simulated data are usually clean and complete, consequently increasing the robustness of the ML model.<br>• The ML model will inherit any errors from the numerical model, and is purely dependent on the generated data<br>• Synthetic data generated may not reflect real scenarios, decreasing generalizability<br>• The ML models still have a semi-blackbox nature if no additional constraints are incorporated<br>• Both the numerical and ML model will need training and testing iteratively if there are any changes that need to be incorporated	ML models could be trained to a wider range of scenarios and climate conditions generated by numerical models. A mix of real-world data and synthetic data can be used for training to increase the robustness and generalizability. Higher resolutions and more defined inputs could be used for simulation and training, however, this tends to increase the computational cost and time.

Type	Description	Advantages and Disadvantages	Future directions
Feature informed ML models (enhanced structure)	ML models can be integrated with important physical and geo-spatial features in the form of high resolution topographic data, hydraulic structures, land use, urban features etc. By embedding detailed information, the model resolutions and coupling strategies are guided and computational efforts are focused on more critical features while simplifying other areas to balance detail and efficiency.	• The realism and representation of the models are increased as physical features are incorporated<br>• Computational efficiency can be increased as the model resolutions can be determined based on features, and consequently computational resources are used more effectively.<br>• Has higher flexibility and better risk assessment capabilities<br>• Data dependent, requiring detailed data of the catchment, therefore are limited in data-scarce areas<br>• High resolution data increases model complexity, and balancing resolution and computational cost becomes challenging<br>• Oversimplification of features that are decided to be less critical may happen	Improvements can be made in the form of incorporating automated feature detection that uses remote sensing data and the ML models automatically extracting and incorporating the data, making the models more useful in real time applications. Multiple modes and sources of data can be used to increase model reliability. Cloud computing could be used to handle data processing and increase scalability and speed of the model.

Type	Description	Advantages and Disadvantages	Future directions
ML models with optimized hyper-parameters (enhanced structure)	Optimization algorithms can be used to optimize the parameters of ML models for increased robustness and accuracy. The hyper-parameters of the model include learning rate, number of layers, batch size, dropout rate and kernel size. There are different methods and architectures of optimization that can be used and evaluated for the most efficient method depending on the requirements.	• Optimized parameters can significantly improve model performance, reduce overfitting and increase robustness<br>• Optimized models can learn from limited data and can work better in data scarce regions<br>• Ensures faster and more stable training, reduced computational waste and increased adaptability across models<br>• Some optimization methods can be expensive and produce black box models that may not generalize well<br>• Over-optimization can lead to overfitting and the model combinations can increase with more hyperparameters	Optimization architectures can be selected depending on the nature of the model and the prediction parameters. Meta-learning can be incorporated to reuse hyperparameters across different locations or flood events. Incorporating hydrological knowledge into the models can make the models less black-box in nature and ensure accuracy.

Type	Description	Advantages and Disadvantages	Future directions
Physics informed neural networks (enhanced structure)	ML models can be trained by enforcing the physics through base ML architectures to solve partial differential equations incorporating a physics informed loss function . They account for the physical laws and constraints for given boundary conditions and have reduced black-box natures.	• Has a reduced need for data due to embedded physics and has improved generalizability<br>• Incorporates hydrodynamic equations into the model increasing physical consistency across data-sparse areas<br>• Can incorporate real-time data into models enabling dynamic now-casting and rapid forecasting<br>• Solving incorporated equations may be computationally intensive for large domains and higher resolutions<br>• Optimization can be difficult and can be limited for complex physics	Hybridizing the PINNs can enhance the speed and accuracy, and balancing techniques can be used to stabilize the training. The domain can be split and trained in parallel to improve scalability and speed.

Type	Description	Advantages and Disadvantages	Future directions
Ensemble data-driven models (enhanced processes)	Multiple ML architectures can be combined to form the different parts of the flood model and to perform different functions (for example one technique to predict outflow and another to predict water depths). Combining models can compensate for the limitations of running a single model by selecting the best model to perform a specific task, consequently adding the advantages. The three common ensemble strategies include bagging (combining outputs from multiple base learners trained on different datasets), boosting (improves predictions by focusing on errors from previous models), and stacking (meta-learner that optimally combines predictions from	• Can lead to better generalization and reduced errors, improving accuracy<br>• Reduces overfitting to specific data characteristics by combining diverse models<br>• Can enhance hybridization by supplementing physics and increase adaptability to working with different types of data<br>• Increases model diversity by combining strengths of different architectures, improving robustness and reliability<br>• Data intensive, requiring high quality and high resolution datasets to achieve optimal performance<br>• Black-box nature can increase by using multiple data driven models, making the understanding of processes difficult<br>• Computationally resource intensive, multiple ML models need training and running on large datasets<br>• Requires careful combination and calibration	Incorporating catchment characteristics and physical constraints can improve model reliability and physical consistency. Real-time data from sensors and radar can be used to continuously update the models, increasing real-time applicability. Techniques can be used to reduce the model complexity without compromising accuracy (model pruning, compression, knowledge distillation). The models best suited for certain tasks can be combined for optimal performance, for example, combining models that specialize in spatial patterns with models best suited for time-series handling.

Type	Description	Advantages and Disadvantages	Future directions
ML models with parallel computing (enhanced processes)	Computational tasks are distributed across multiple processes or cores to perform simulations simultaneously. In hybrid models with different modeling approaches, parallel computing manages the complexity by dividing the workload and speeds up the model. Commonly used in large-scale high-resolution models.	• Accelerates simulations by performing multiple calculations simultaneously, enabling rapid forecasting<br>• Allows models to handle larger domains and finer spatial resolutions, consequently improving scalability<br>• More details can be incorporated into models, such as detailed physics, where multi-core CPUs and GPUs can optimize hardware capabilities.<br>• Can be challenging to design efficient parallel algorithms to work concurrently<br>• Data exchange between components or processors can create lags, compromising efficiency<br>• Processors need balanced and even tasks to be assigned for optimal performance<br>• Can be hardware dependent, and knowledge of the hardware components are essential	Improvements can be made by implementing adaptive strategies to distribute tasks dynamically and evenly during the processing stage, maximizing the output. Hybrid parallelization can be done, for example combining distributed memory and shared memory (MPI and OpenMp) for increased flexibility and performance. Hardware accelerators can be leveraged and the breakdown of tasks can be evaluated and implemented.

Type	Description	Advantages and Disadvantages	Future directions

Table 6 lists the hybrid models presented in literature with different hybridization techniques. Some models are developed with combined forms of hybridization which was also taken into account, and are mentioned separately in each method incorporated. The list was generated from about 50% of the reviwed literature for simplicity.

Table 6: Models presented in literature categorized according to the methods of hybridization in flood inundation models

**Hybridization method**	**Models presented**
ML models incorporating SAR data (Enhanced input)	• Ngo et al. (2021) presented an optimized ML (QPSO-CDTreeEns) model by using SAR data to build the Credal Decision Treee (CDT) model for spatial prediction of floods in the Tran Yen District of Vietnam.<br>• Ngo et al. (2018) used SAR data to train an ensemble ML model (FA-LM-ANN) in the Bac Ha Bao Yen district, Vietnam for spatial prediction of flash floods.<br>• Liu et al. (2019) used SAR data to build the SARCFMNet - CNN model for coastal inundation mapping for hurricane Harvey in Texas, USA

**Hybridization method**	**Models presented**
ML models incorporating hydrodynamic models (Enhanced input)	• Kabir et al. (2020) used a hydraulic model to train a CNN model for rapid fluvial flood inundation modeling the city of City of Carlisle, UK.<br>• Liao et al. (2023) used a coupled model based on physical mechanisms to generate a rainfall-inundation database to train a MORF model aimed at building a framework for rapid urban flood modeling in the Chebei River Basin, Guangzhou City, China.<br>• Li and Willems (2020) used lumped physics based models to generate hydrological responses as input into a Multivariate Logistic Regression model for urban pluvial flood prediction in Antwerp and Gent, Belgium.<br>• Oliveira et al. (2022) used continuously running conventional numerical models to detect water level thresholds that trigger the execution of ML based simulations using real-time monitoring for flash flood forecasting in the Ribeira das Vinhas basin, Cascais.<br>• Tewari et al. (2021) used an LSTM model alongside a Height Above Nearest Drainage (HAND) model to predict river stage in real-time and generate inundation area maps in Cedar Rapids, Iowa, US.<br>• Dang et al. (2024) used numerical models to train and compare several ML models for flood depth prediction in the Ho Chi Minh City, Vietnam.<br>• Xie et al. (2021) used a 2D hydrodynamic model to train several ANN models for flood level prediction of the Burnett River in Queensland, Australia.<br>• Jhong et al. (2022) used a numerical hydrodynamic model to simulate inundation depth data for training and testing a adaptive neuro-fuzzy inference system, support vector machine, and an ensemble model combining support vector machine and a multi-objective genetic algorithm for assessing the effective spatial characteristics of input features in the Yilan County, Taiwan.

**Hybridization method**	**Models presented**
Targeted optimization of models (Enhanced structure)	• Ngo et al. (2021) used Quantum Particle Swarm Optimization (QPSO) to optimize the three parameters, the subspace size, number of trees, and the maximum depth of trees of the QPSO-CDTreeEns model.<br>• Tien Bui et al. (2019) used a GIS database was to build and verify a Multivariate Adaptive Regression Splines (MARS) prediction model incorporating Particle Swarm Optimization (PSO) for spatial prediction of flash flooding in Antwerp and Gent, Belgium.<br>• Rahmati et al. (2020) compared two methods of optimization combined with an ML model, Wavelet-SVR-Bat and Wavelet-SVR-GWO, for spatial modeling of urban flood susceptibility city of Amol, Iran.

**Hybridization method**	**Models presented**
Feature informed ML models (Enhanced structure)	• Schmid and Leandro (2023) used a hybrid database with pre-simulated scenarios is used to train, validate, and test a CNN model with feature informed dense layers for a flood forecast system in Kulmbach, Bavaria, Germany.<br>• Situ et al. (2025) used a LSTM-SegNet-MSA with deep fusion of spatial and temporal features for rapid prediction of flood dynamics in Hohhot, China.<br>• Tripathi and Mohanty (2024) forced geomorphic flood descriptors (GFDs) into a series of ML models to build a framework for estimating flood hazard and population exposure in the Ganges Basin, India<br>• Adriano et al. (2023) accounted for topographical information when training the CNN model for flood inundation mapping in four the four regions Hijikawa, Omachi, Okayama, and Ibaraki in Japan
Physics informed ML models (Enhanced structure)	• Donnelly et al. (2023) presented a CNN based PINN model as a surrogate model for the hydrodynamic simulator (Delft3D) for the English Channel.<br>• Balakrishna Madayala et al. (2022) used hydrological model data, synthetic data and observed data was used to train an ANN-based PINN for urban flood modelling in the Uttar Pradesh in Northern India.

**Hybridization method**	**Models presented**
Ensemble ML models (Enhanced processing)	• Xu and Gao (2024) combined a long short-term memory (LSTM) neural network for predicting drainage outflows with a one-dimensional convolutional neural network (1D CNN) for predicting water depths in a LSTM-CNN model used for real-time flooding prediction that considers the compound effects of storm tides, rainfall, and drainage outflows in Macao, China.<br>• Kabir et al. (2021) built a rainfall–discharge model based on random forest (RF) technique on top of classifiers based on multi-layer perceptron (MLP) to generate probabilistic flood inundation maps for real-time applications in Worcestershire, England.<br>• Ngo et al. (2018) presented a combined firefly algorithm, Levenberg–Marquardt backpropagation, and an artificial neural network (FA-LM-ANN) model for spatial prediction of floods in Bac Ha Bao Yen, Vietnam.<br>• Ebtehaj and Bonakdari (2022) presented a discrete wavelet transform (DWT) and improved outlier-robust extreme learning machine (IORELM) ensemble model DWTIORELM, for real-time multi-steps-ahead flood forecasting in the Saint-Lawrence River in Quebec.<br>• Jhong et al. (2022) presented a hybrid model combining support vector machine and a multi-objective genetic algorithm to assess the effective spatial characteristics of input features in Yilan county, Taiwan.<br>• Chang et al. (2018) built a combined self-organizing map (SOM) and a recurrent nonlinear autoregressivewith exogenous inputs (RNARX) for generating real-time regional flood inundation maps for the Kemaman River Basin in Malaysia.<br>• Chang et al. (2010) presented a clustering-based hybrid inundation model (CHIM) based on Linear regression and BPNN to generate 1-h ahead flood inundation maps for Dacun Township, Taiwan.<br>• Sharma and Saharia (2025) built a CNN and Vision Transformer (ViT) model trained on an weak flood [illegible]

Hybridization method	Models presented
Parallelization of processing (Enhanced processing)	• Dang et al. (2024) trained nine ML models on single processing framework for one domain and enabling parallel processing for eight domains noting that parallel processing was about 5.4 times faster (Ho Chi Minh City, Vietnam).<br>• Sharma and Saharia (2025) proposed a methodology for generating SAR based maps using DL models trained on weak flood labels generated from concurrent optical imagery iswhich was fully automated and parallelized and allowing for optimized training data generation (Pakistan floods of 2022 and Assam floods of 2020 in India).

7.2. Metrics for evaluating flood models

A vast number of metrics can be used for evaluating flood inundation models, and Table 7 shows the popular methods derived from the articles reviewed. The reference values can either be observed, generated from a numerical model, database values or from SAR data. The simulated values refer to the values generated by the hybrid flood model. The parameters range from flow values, flood depth, extent, flow velocity etc.

Table 7: Performance metrics used for evaluation of hybrid flood inundation models (Extended from Table 2)

**Metric**	**Equation**	**Description**	**References**
Coefficient of determination (R^2)	$$R^2 = 1 - \frac{\sum_{i=1}^{n}(V_{\text{ref},i} - V_{\text{sim},i})^2}{\sum_{i=1}^{n}(V_{\text{ref},i} - \overline{V}_{\text{ref}})^2} \quad (1)$$ where: • $V_{\text{ref},i}$ is the reference value at time step i, • $V_{\text{sim},i}$ is the simulated value at time step i, • $\overline{V}_{\text{ref}}$ is the mean of reference values, • n is the total number of time steps.	Is a common performance metric used to evaluate the variability of the data. It can be used as a measure in model calibration for determining the similarity to reference data, in model validation to evaluate how well models can predict new reference data, and in model comparison for analyzing the similarity between two models	Dang et al. (2024), Bentivoglio et al. (2022), Chang et al. (2014), Sirsant et al. (2024), Mosavi et al. (2018), Liao et al. (2023), Zanchetta and Coulibaly (2022), Jhong et al. (2022)

Metric	Equation	Description	References
Mean absolute error (MAE)	$$\mathrm{MAE} = \frac{1}{n}\sum_{i=1}^{n} \lvert V_{\mathrm{ref},i} - V_{\mathrm{sim},i} \rvert \quad (2)$$	Measures the average error values between reference and predicted data without considering if the model overestimates or underestimates the values. A lower MAE is tied to better model performance, indicating that predicted and reference values are close. Usually used for continuous predicitons.	Bentivoglio et al. (2022), Sun et al., Jhong et al. (2018), Liao et al. (2023), Kabir et al. (2021), Jhong et al. (2022), Xu and Gao (2024)

Metric	Equation	Description	References
Median absolute deviation (MAD)	$$\text{MAD} = \text{median}\left(\lvert x_i - \text{median}(x)\rvert\right) \quad (3)$$ where: • x_i represents each value in the dataset (e.g., errors or reference values), • $\text{median}(x)$ is the median of the dataset, • The absolute differences are taken between each value and the median.	Measures the dispersion or variability between residual values, however it uses the median for calculations in contrast to the MAE, making it more robust to outliers. Commonly used when the dataset contains outliers or is not distributed, and is resistant to extreme events.	Dang et al. (2024)
Maximum error (MaxE)	$$\text{MaxE} = \max\left(\lvert V_{\text{ref},i} - V_{\text{sim},i}\rvert\right) \quad (4)$$	Depicts the largest absolute error between reference and predicted values, identifying the worst case error. Useful in models where extreme deviations can be critical.	Dang et al. (2024)

Metric	Equation	Description	References
Mean absolute percentage error (MAPE)	$$\text{MAPE} = \frac{100}{n} \sum_{i=1}^{n} \left| \frac{V_{\text{ref},i} - V_{\text{sim},i}}{V_{\text{ref},i}} \right| \quad (5)$$ where: • n is the total number of references. [nosep, leftmargin=*]	Measures the average magnitude of errors in predictions as a percentage of the actual reference values and indicates the variation of the predicted values from the reference values. Commonly used for interpretable model errors, but can be limited when reference values are close to zero. The model performance increases with decreasing MAPE.	Xu et al. (2023)
Sum of squared errors (SSE)	$$\text{SSE} = \sum_{i=1}^{n} (V_{\text{ref},i} - V_{\text{sim},i})^2 \quad (6)$$	Quantifies the total deviation between reference and predicted values by summing the squares of the errors at each time step or location. Creates the basis for other error metrics. Performance increases with decreasing SSE.	Xu et al. (2023), Ebtehaj and Bonakdari (2022)

Metric	Equation	Description	References
Root mean square error (RMSE)	$$\text{RMSE} = \sqrt{\frac{1}{n}\sum_{i=1}^{n}\left(V_{\text{ref},i} - V_{\text{sim},i}\right)^2} \quad (7)$$	Measures the square root of the average squared differences between reference data and model data, however, it is more sensitive to larger errors as it squares the values to give extra weight before averaging. RMSE emphasizes outliers and is a very common performance metric used in flood modeling. Performance increases with decreasing RMSE.	Yang et al. (2016), Xu et al. (2023), Dang et al. (2024), Sirsant et al. (2024), Chang et al. (2014), Xu et al. (2023), Liao et al. (2023), Kabir et al. (2021), Kabir et al. (2020), Zanchetta and Coulibaly (2022), Jhong et al. (2022), Schmid and Leandro (2023), Chang et al. (2010), Wijaya [illegible]

Metric	Equation	Description	References
Mean squared error (MSE)	$$\text{MSE} = \frac{1}{n}\sum_{i=1}^{n}(V_{\text{ref},i} - V_{\text{sim},i})^2 \quad (8)$$	Measures the average of the squared differences between predicted and reference values. Commonly used to evaluate continous values such as water levels. Performance increases when MSE decreases.	Schmid and Leandro (2023), Xu and Gao (2024)
Relative root mean square error (RRMSE)	$$\text{RRMSE} = \frac{\text{RMSE}}{\overline{V_{\text{ref}}}} \times 100 \quad (9)$$	Similar to RMSE, however, it normalizes values making it easier to use in comparison and expresses the RMSE as a percentage of the mean observed values. As the RMSE and RRMSE decreases, model performance increases.	Xie et al. (2021)

Metric	Equation	Description	References
Nash suttcliffe efficiency (NSE)	$$\text{NSE} = 1 - \frac{\sum_{i=1}^{n} \left(V_{\text{ref},i} - V_{\text{sim},i}\right)^2}{\sum_{i=1}^{n} \left(V_{\text{ref},i} - \overline{Q_{\text{ref}}}\right)^2} \quad (10)$$	Evaluates how well a the model predicts observed data by comparing the variance of the prediction model errors to the variance of the reference data. The NSE, also known as the coefficient of efficiency, considers both timing and magnitude into analysis, and an NSE of 1 indicates a perfect match between predicted and reference data	Xu et al. (2023), Mosavi et al. (2018), Xie et al. (2021), Kabir et al. (2021), Kabir et al. (2020), Jhong et al. (2022), Chen et al. (2022), Xu and Gao (2024)

**Metric**	**Equation**	**Description**	**References**
Accuracy	$$\text{Accuracy} = \frac{TP + TN}{TP + TN + FP + FN} \quad (11)$$ where: • TP = True Positives • TN = True Negatives • FP = False Positives • FN = False Negatives	Accuracy can be depicted in multiple metrics, and determines how similar the reference and predicted values are. Refers to the proportion of correct predictions by the model relative to the total number of predictions. High accuracy indicates better performance.	Bentivoglio et al. (2022), Yang et al. (2016)
Precision	$$\text{Precision} = \frac{\text{TP}}{\text{TP} + \text{FP}} \quad (12)$$	Is a measure of the events that are correctly predicted. Commonly used for predicting binary events such as in maps that determine flood and non-flooded areas. Multiple metrics can be used to define precision.	Li and Willems (2020), Tien Bui et al. (2019), Ngo et al. (2018)

Metric	Equation	Description	References
Recall	$$\text{Recall} = \frac{\text{TP}}{\text{TP} + \text{FN}} \quad (13)$$	Is closely related to precision, and is also known as sensitivity or true positive rate. It measures how well a model identifies actual flood events. As precision and recall increases, the model performance improves.	Sirsant et al. (2024), Kabir et al. (2020), Li and Willems (2020), Xu and Gao (2024), Ngo et al. (2018)
F1 score	$$\text{F1 Score} = \frac{2 \times \text{TP}}{2 \times \text{TP} + \text{FP} + \text{FN}} \quad (14)$$	Is the harmonic mean of precision and recall, and balances the tradeoff between fasle positives and false negatives. Useful in flood detection models were both types of errors are costly. F1 = 1 indicates perfect precision and recall.	Sirsant et al. (2024), Bentivoglio et al. (2022), Kabir et al. (2021), Li and Willems (2020), Xu and Gao (2024)

Metric	Equation	Description	References
Overall accuracy (OA)	$$\text{OA} = \frac{\text{TP} + \text{TN}}{\text{TP} + \text{FP} + \text{FN} + \text{TN}} \quad (15)$$	Measures the proportion of correct predictions (flood and no-floods) over the total number of cases.	Panahi et al. (2022), Ngo et al. (2018)
Producer accuracy (PA)	$$\text{PA} = \frac{\text{TP}}{\text{TP} + \text{FN}} \quad (16)$$	The probability that a reference flood event was correctly classified by the model. Equivalent to sensitivity and is useful in evaluating the ommision error.	Panahi et al. (2022)
User accuracy (UA)	$$\text{UA} = \frac{\text{TP}}{\text{TP} + \text{FP}} \quad (17)$$	The probability that a predicted flood event actually coresponds to a real flood. Equavalant to precision in classififcation and reflects the commision error (false alarms).	Panahi et al. (2022)

Metric	Equation	Description	References
Probability of detection (POD)	$$\text{POD} = \frac{\text{TP}}{\text{TP} + \text{FN}} \quad (18)$$	Measures the model's ability to correctly predict flood occurrences where flooding actually happened.	Yang et al. (2016), Xie et al. (2021), Zanchetta and Coulibaly (2022), Sun et al., Chen et al. (2022)
False alarm ratio (FAR)	$$\text{FAR} = \frac{\text{FP}}{\text{TP} + \text{FP}} \quad (19)$$	Measures the proportion of predicted flood locations by the model that were not actually flooded. It indicates how often the model raises false alarms by incorrectly predicting flooding.	Yang et al. (2016), Xie et al. (2021), Zanchetta and Coulibaly (2022), Sun et al., Chen et al. (2022)

Metric	Equation	Description	References
Success ratio (SR)	$$\mathrm{SR} = \frac{\mathrm{TP}}{\mathrm{TP} + \mathrm{FP}} \quad (20)$$	Measures the fraction of predicted flood locations that were actually flooded.	Yang et al. (2016), Xie et al. (2021), Zanchetta and Coulibaly (2022), Sun et al., Chen et al. (2022)
Threat score (TS also known as critical success index)	$$\mathrm{TS} = \frac{\mathrm{TP}}{\mathrm{TP} + \mathrm{FP} + \mathrm{FN}} \quad (21)$$	Measures the fraction of predicted flood locations that were actually flooded. It balances hits against both misses and false alarms, providing a comprehensive measure of model accuracy in flood detection. TS ranges from 0 (no skill) to 1 (perfect prediction).	Yang et al. (2016), Xie et al. (2021), Zanchetta and Coulibaly (2022), Sun et al., Chen et al. (2022)

Metric	Equation	Description	References
Classification accuracy rate	$$\text{Accuracy} = \frac{\text{TP} + \text{TN}}{\text{TP} + \text{TN} + \text{FP} + \text{FN}} \quad (22)$$	Measures the proportion of correctly classified instances (or predictions) over the total number of instances. It reflects the overall accuracy of a flood inundation model in distinguishing flooded versus non-flooded areas.	Tien Bui et al. (2019)
Bias	$$\text{Bias} = \frac{\text{Total Predicted Flooded}}{\text{Total Reference Flooded}} = \frac{\text{TP} + \text{FP}}{\text{TP} + \text{FN}} \quad (23)$$	Measures the tendency of a model to systematically overpredict or underpredict flood occurrences or magnitudes. 1 indicates perfect agreement and higher values indicate overprediction.	Zanchetta and Coulibaly (2022)
Matthews corelation coefficient	$$\text{MCC} = \frac{\text{TP} \times \text{TN} - \text{FP} \times \text{FN}}{\sqrt{(\text{TP} + \text{FP})(\text{TP} + \text{FN})(\text{TN} + \text{FP})(\text{TN} + \text{FN})}} \quad (24)$$	Is a balanced measure of the quality of binary classifications, accounting for all four categories in a confusion matrix which are TP, TN, FP, and FN. -1 indicates total disagreement, 0 indicates random, and 1 indicates perfect prediction.	

Metric	Equation	Description	References
The receiver operating characteristic curve (ROC)	$$\text{TPR} = \frac{\text{TP}}{\text{TP} + \text{FN}} \quad \text{versus} \quad \text{FPR} = \frac{\text{FP}}{\text{FP} + \text{TN}} \quad (25)$$	ROC is a graphical plot that shows the diagnostic binary classifer system as it's discrimination threshold is varied (how much a model can differentiate between positive and negative classes)	Bentivoglio et al. (2022), Tien Bui et al. (2019), Ngo et al. (2018)
Speed up	$$\text{Speed} = \frac{\text{Total Distance or Area Processed}}{\text{Processing Time}} \quad (26)$$ where: • Total Distance or Area Processed = extent of flood area or model domain processed, • Processing Time = time taken by the model to produce predictions.	Is a point of comparison for surrogate models and is the ratio between the simulation time of the numerical and novel model. The comparison of speed up of models depend on the number of numerical simulations and type of model, with similar discrepancies arising in error scores, which in turn depend on the scale of the case study, resolution and the underlying numerical simulator.	Bentivoglio et al. (2022)

Metric	Equation	Description	References
Megacells per scond	$$\text{Speed} = \frac{\text{Number of Processed Cells}}{\text{Processing Time} \times 10^6} \quad \text{(megacells per second)} \tag{27}$$ where: • Number of Processed Cells = total grid cells computed by the model, • Processing Time = time taken (in seconds) to process these cells.	Compares the computational efficiency across all implementations (number of cells per computational time). Helpful in evaluating the computational efficiency and real-time applicability of the model.	Marshall et al. (2017)
I/O seconds	$$\text{I/O Time} = t_{\text{read}} + t_{\text{write}} \tag{28}$$ where: • t_{read} = time spent reading input data (seconds), • t_{write} = time spent writing output data (seconds).	Refers to the time that is spent specifically on data reading and writing to disk and the MPI communications (input and output operations). Smaller I/O time indicates higher efficiency.	Marshall et al. (2017)

**Metric**	**Equation**	**Description**	**References**
structural similarity index (SSIM)	$$\text{SSIM}(x, y) = \frac{(2\mu_x\mu_y + C_1)(2\sigma_{xy} + C_2)}{(\mu_x^2 + \mu_y^2 + C_1)(\sigma_x^2 + \sigma_y^2 + C_2)} \quad (29)$$ where: • x and y = the two images being compared, • μ_x, μ_y = mean intensities of x and y, • σ_x^2, σ_y^2 = variances of x and y, • σ_{xy} = covariance of x and y, • C_1, C_2 = constants to stabilize the division (typically small values).	Determines the perceptual similarity between two images generated, often to compare simulated flood inundation maps with reference flood maps. 1 indicates perfect similaity and -1 indicates no similarity.	Adriano et al. (2023)

Metric	Equation	Description	References
Error to time of peak discharge (ETP)	$$\text{ETP} = \lvert t_{\text{peak, reference}} - t_{\text{peak, modeled}} \rvert \quad (30)$$ where: • $t_{\text{peak, reference}}$ = time of peak discharge from reference data, • $t_{\text{peak, modeled}}$ = time of peak discharge simulated by the model.	Quantifies the deviation of the predicted peak discharge time from the reference peak discharge time (usually at the peak). It quantifies the timing accuracy of the model in predicting peak flod and is critical for flood warning and management.	Jhong et al. (2018)
The Kappa score	$$\kappa = \frac{p_o - p_e}{1 - p_e} \quad (31)$$ where: • p_o = observed agreement (proportion of times the model and references agree), • p_e = expected agreement by chance (calculated from marginal totals).	Measures the agreement between two categorical variables, taking the agreement occurring by chance into account. It evaluates how well the models flood predictions agree with reference data beyond random chance. -1 indicates complete disagreement and 1 indicates perfect agreement.	Ngo et al. (2021)

Metric	Equation	Description	References
Pearson correlation coefficient (PCC)	$$r = \frac{\sum_{i=1}^{n}(x_i - \bar{x})(y_i - \bar{y})}{\sqrt{\sum_{i=1}^{n}(x_i - \bar{x})^2}\sqrt{\sum_{i=1}^{n}(y_i - \bar{y})^2}} \quad (32)$$ where: • x_i and y_i = paired data points (e.g., reference and modeled values), • $\bar{x}$ and $\bar{y}$ = mean of x and y, • n = number of paired references.	Measures the linear relationship between continuous variables such as the predicted and reference time-series. -1 indicates perfect negative correlation,, 0 indicates no correlation and 1 perfect positive positive correlation.	Liao et al. (2023)
Normalized Bias (NB)	$$\mathrm{NB} = \frac{\sum_{i=1}^{n}(P_i - O_i)}{\sum_{i=1}^{n} O_i} \quad (33)$$ where: • P_i = simulated value at location i, • O_i = reference value at location i, • n = total number of locations or data points.	Quantifies the relative bias between modeled and observed value sand is normalized by the observed total. It shows the degree to which the model systematically over- or under-predicts, expressed as a fraction or percentage, where 0 indicates no bias.	Chen et al. (2022)

Metric	Equation	Description	References
Friedman test	$$\chi_F^2 = \frac{12}{nk(k+1)} \sum_{j=1}^{k} R_j^2 - 3n(k+1) \quad (34)$$ where: • n is the number of blocks (e.g., flood events), • k is the number of treatments (e.g., models), • R_j is the sum of the ranks for treatment j across all blocks.	Non-parametric statistical test used to detect differences in treatments across multiple test attempts. Compares the performance of several flood models or algorithms over multiple datasets when the assumptions of parametric tests are not met.	Marshall et al. (2017), Sarwar et al. (2024)

Metric	Equation	Description	References
Wilcoxon signed rank test (non-parametric)	**Steps:**<br>1. Compute the differences: $d_i = x_i - y_i$ for all paired reference values.<br>2. Discard pairs with $d_i = 0$.<br>3. Rank the absolute differences $\|d_i\|$, assigning average ranks in case of ties.<br>4. Assign the sign of d_i to each rank.<br>5. Compute the test statistic:<br>$$W = \min\left(\sum_{\text{positive ranks}} R_i, \sum_{\text{negative ranks}} R_i\right)$$	Compares the performance of two models on a pairwise basis across the same set of events	Marshall et al. (2017)
